# Impact of meditation on brain age derived from multimodal neuroimaging in experts and older adults from a randomized trial

**DOI:** 10.1038/s41598-025-21490-9

**Published:** 2025-10-28

**Authors:** Sacha Haudry, Natacha Lambert, Christian Gaser, Bertrand Thirion, Brigitte Landeau, Julie Gonneaud, Géraldine Poisnel, Pierre Champetier, Asrar Lehodey, Natalie L. Marchant, Olga Klimecki, Fabienne Collette, Denis Vivien, Vincent de la Sayette, Antoine Lutz, Gaël Chételat

**Affiliations:** 1 Normandy University, UNICAEN, INSERM, UA20, Neuropresage, Cyceron, 14000 Caen, France; 2 GRAYC Laboratory, University of Caen Normandy, Caen, France; 3 Department of Neurology, Jena University Hospital, Jena, Germany; 4 Department of Psychiatry and Psychotherapy, Jena University Hospital, Jena, Germany; 5 German Center for Mental Health (DZPG), Jena, Germany; 6 Inria, CEA, Université Paris-Saclay, Paris, France; 7 Division of Psychiatry, Faculty of Brain Sciences, University College London, London, UK; 8 Developmental Psychology, Friedrich-Schiller-Universität Jena, Jena, Germany; 9 Biological Psychology, Faculty of Psychology, Technische Universität Dresden, Dresden, Germany; 10 GIGA-CRC Human Imaging, University of Liège, Liège, Belgium; 11 Psychology and Neuroscience of Cognition Research Unit, University of Liège, Liège, Belgium; 12 Normandy University, UNICAEN, INSERM, U1237, PhIND “Physiopathology and Imaging of Neurological Disorders”, Institut Blood & Brain @ Caen, Cyceron, 14000 Caen, France; 13 Département de Recherche Clinique, CHU Caen-Normandie, Caen, France; 14 Service de Neurologie, CHU de Caen, Caen, France; 15 Lyon Neuroscience Research Center, INSERM U1028, CNRS UMR5292, Lyon 1 University, Lyon, France

**Keywords:** Brain imaging, Neural ageing, Learning algorithms, Neuroscience, Health care

## Abstract

Meditation is thought to promote healthy aging by improving mental health, preserving brain integrity and reducing Alzheimer’s disease risk. We examined the impact of long-term meditation expertise and an 18-month meditation training on brain aging in older adults using machine learning. We included 25 Older Expert Meditators (OldExpMed) with > 20 years of practice and 135 Cognitively Unimpaired Older Adults (CUOA) from the Age-Well randomized controlled trial. CUOA were randomized (1:1:1) into an 18-month meditation training, a non-native language training, and a no intervention group. Brain age was predicted using a machine learning model trained on gray and white matter volume and glucose metabolism data from ADNI and replicated with a second model. Brain Predicted Age Difference (BrainPAD) was computed as the gap between predicted and chronological age. We assessed meditation expertise effects on BrainPAD, its links with meditation hours, cognitive, and affective measures, and the impact of 18-month training. Compared to CUOA, OldExpMed exhibited significantly lower/more negative BrainPAD, linked to meditation hours, mental imagery, and prosocialness. No significant effect of 18-month training was observed. Results were consistent across the replication model. Long-term meditation is associated with younger brain age, but 18-month training has no effect, emphasizing the need for sustained practice to support healthy brain aging.

**Supplementary Information:**

The online version contains supplementary material available at 10.1038/s41598-025-21490-9.

## Introduction

Population aging has been accelerating rapidly in recent years and will continue to do so in the near future. As the number of older adults increases, ensuring healthy aging has become a critical priority. This is particularly important given that depression remains the most common psychiatric disorder in people over 65, with prevalence estimates ranging from 10 to 30%^1,2^. Moreover, depressive symptoms are frequently underdiagnosed or overlooked in 60 to 70% of cases in this age group^3^. These conditions—including depression, anxiety, and stress—contribute to deteriorating mental health and elevate the risk of developing dementia. Meditation, a set of complex emotional and attentional regulatory strategies aimed at enhancing well-being, reducing stress, and promoting emotional balance^4,5^, may offer a promising solution. By mitigating these psycho-affective factors, meditation could support healthy aging and potentially lower the risk of dementia^6^. In this context, research has explored the effects of both meditation interventions and meditation expertise through cross-sectional or longitudinal designs^7–20^. Such studies have demonstrated positive effects of meditation on cognition—particularly in attention and executive functions^7–9,21^—as well as on psycho-affective factors such as depression, anxiety, and stress^10–15^. Additionally, meditation has been linked to enhanced brain integrity, notably in regions vulnerable to Alzheimer’s disease^15–17^. However, while research on expert meditators—who have practiced for years or even decades—consistently shows more preserved brain structure and function compared to non-meditators, findings from longitudinal studies on meditation interventions (e.g., 8 weeks to 18 months) are limited and inconsistent. These studies report a range of outcomes in adults and older populations, from positive effects^22–25^ to no effects^20,26^, and even negative ones^19^. In the Age-Well clinical trial, part of the European Medit-Ageing project and the only study to date to include multimodal neuroimaging with an 18-month meditation training in cognitively unimpaired older adults (CUOA), we found no significant effects of meditation on the volume or perfusion of the anterior cingulate and insula compared to passive and active control groups^20^. In the present study, instead of focusing solely on specific brain structures, we aimed to employ a more integrative, bottom-up, and comprehensive strategy that takes into account the entire brain and multiple imaging modalities. This method has gained substantial traction in aging research as a sensitive marker of brain integrity, and its deviation from chronological age (brain-PAD) has been linked to cognition, mortality, and risk for neurodegenerative diseases^27,28^. Machine learning techniques have the potential to analyze comprehensive brain imaging data across various modalities, thereby uncovering more complex patterns of cerebral integrity and meditation effects than traditional methods typically allow. To our knowledge, only two studies have used machine learning to investigate the effects of meditation by estimating cerebral age based on neuroimaging^29,30^. These studies showed that expert meditators had a lower predicted brain age compared to meditation-naive controls. However, both existing studies relied on a single imaging modality, as they used solely Gray Matter Volume (GMV) maps for brain prediction which limits the information available to the model and may reduce its precision. Moreover, neither study specifically focused on older expert meditators, as the first included a single participant aged 41 years old, while the second examined expert meditators with a wide age range (24–77 years). Finally, no study to date has explored this question longitudinally in the context of a meditation intervention.

In the present study, we investigated the impact of meditation on brain integrity using an integrative machine learning approach to predict brain age from multimodal neuroimaging data and estimate Brain Predicted Age Difference (BrainPAD), which reflects the gap between predicted brain age and chronological age. We assessed the impact of meditation on BrainPAD both cross-sectionally, comparing Older Expert Meditators (OldExpMed) to controls, and longitudinally, following an 18-month meditation training in meditation-naive CUOA. Additionally, we explored whether any observed effect on brain aging was associated with accumulated meditation hours, cognitive performance and measures of affective regulation capacity. Voxelwise correlation analyses were also conducted to identify brain regions most contributing to brain age predictions. To ensure robustness, all analyses were replicated with an independent brain age prediction model trained on a separate dataset. We hypothesized that both meditation expertise and the 18-month meditation training would be associated with slower brain aging (i.e., a lower BrainPAD) compared to controls, and that this effect would correlate with longer practice, better cognitive performance and improved emotional balance.

## Results

Demographic characteristics of participants included in the cross-sectional and longitudinal analyses are summarized in Table 1. Excellent participant compliance with the intervention was observed, and no serious adverse events were registered in any of the three groups^20^. All results described below are those obtained with the main model. The results obtained with the replication model are detailed in the Supplementary materials (Supplementary results) and summarized at the end of the result section.

**Table 1:** Demographics of the participants.

Characteristics	Meditation training group	Non-native language training group	No Intervention group	OldExpMed group	CUOA group
Sample size	45	44	45	25	135
Age	69.4 ± 3.69 (65–78)	70.3 ± 4.55 (65–84)	68.2 ± 2.75 (65–75)	70.3 ± 4.53 (65–85)	69.3 ± 3.8 (65–84)
Education	13.1 ± 3.07 (7–22)	12.2 ± 3.06 (7–17)	14.2 ± 2.87 (7–20)	15.16 ± 3.46 (9–24)	13.16 ± 3.09 (7–22)
Sex M/F (%)	14/31 (31.1/68.9)	20/24 (45.5/54.5)	18/27 (40/60)	16/9 (64/36)	52/83 (38.5/61.5)
MMSE	28.93 ± 1.18 (26–30)	28.95 ± 1.01 (27–30)	29.22 ± 0.89 (27–30)	29.04 ± 1.03 (27–30)	29.04 ± 1.03 (26–30)
Hours of meditation practice	N.A	N.A	N.A	32,501 ± 31,120 (10,184–164,250)	0

Values are mean SD (range) unless specified otherwise. No statistical tests were conducted to compare baseline demographic characteristics between groups. Given the randomization procedure, no significant differences were expected, and no major clinical differences in demographics were observed between groups. OldExpMed, older expert meditators, CUOA, cognitively unimpaired older adults, MMSE, mini mental state examination, N.A, not applicable.

### Evaluation of the model

The main model was trained using GMV, white matter volume (WMV), and Fluorodesoxyglucose-Positron Emission Tomography (FDG-PET) maps of 320 cognitively unimpaired elderly individuals (age range: 60–96 years old) from the Alzheimer’s Disease Neuroimaging Initiative (ADNI) dataset and applying Lasso regression. It successfully predicted chronological age in the test dataset (predicted-chronological age correlation: *p* < 0.001, β = 0.37, Mean Absolute Error [MAE] = 3.8) (Supplementary Fig. S1A).

### Assessment of covariates

Sex and education significantly differed between OldExpMed and meditation-naive CUOA (sex: *p* = **0.003**, x^2^ = 8.74 ; male/female frequencies – OldExpMed: 15/5, CUOA: 33/59 & education: *p* = **0.01**, t = 2.71; mean years of education – OldExpMed: 15.15, CUOA: 12.74) while there was no significant difference for age (*p* = 0.14, t = 1.52 ; mean age – OldExpMed: 70.5, CUOA: 68.82). Moreover, there was no significant interaction between group and any of the covariates on BrainPAD (age: *p* = 0.39, sex: *p* = 0.73, education: *p* = 0.07). As a result, both sex and education were included as covariates in the OldExpMed versus CUOA comparison analysis. Additionally, in the OldExpMed group, no significant association was found between BrainPAD and education (*p* = 0.93, β = 0.02) nor was there any difference in BrainPAD between males and females (*p* = 0.85, β = 0.42). However, age was significantly associated with BrainPAD (*p* = **0.002**, β = 0.73), so it was included as a covariate in the regression analyses within the OldExpMed group. In the meditation training group, no significant association was found between the longitudinal evolution of BrainPAD (ΔBrainPAD) and age (*p* = 0.1, β = − 0.06), sex (*p* = 0.43, β = 0.17) nor education (*p* = 0.85, β =− 0.01), thus no covariate was included in the analyses within the meditation training group.

### Difference in BrainPAD between OldExpMed and meditation-naive CUOA

**Fig. 1:**
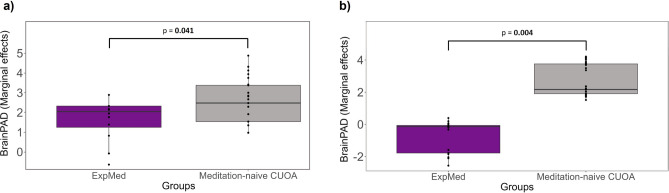
Comparison of BrainPAD between OldExpMed and CUOA. The boxplots show the difference of BrainPAD between OldExpMed (purple) and meditation-naive CUOA (gray) (**a**) with the main model and (**b**) with the replication model. Marginal effects were plotted to adjust for the effects of sex and education. Horizontal lines represent the median of each group, open boxes show the 25^th^ and 75^th^ percentile, and vertical lines show data range. BrainPAD, brain predicted age difference; OldExpMed, older expert meditators; CUOA, cognitively unimpaired older adults.

OldExpMed had a significantly lower/more negative BrainPAD compared to CUOA (F = 4.26, *p* = **0.041**). The between-group difference is illustrated in Fig. 1A.

### Links between BrainPAD and the accumulated hours of meditation practice in OldExpMed

**Fig. 2:**
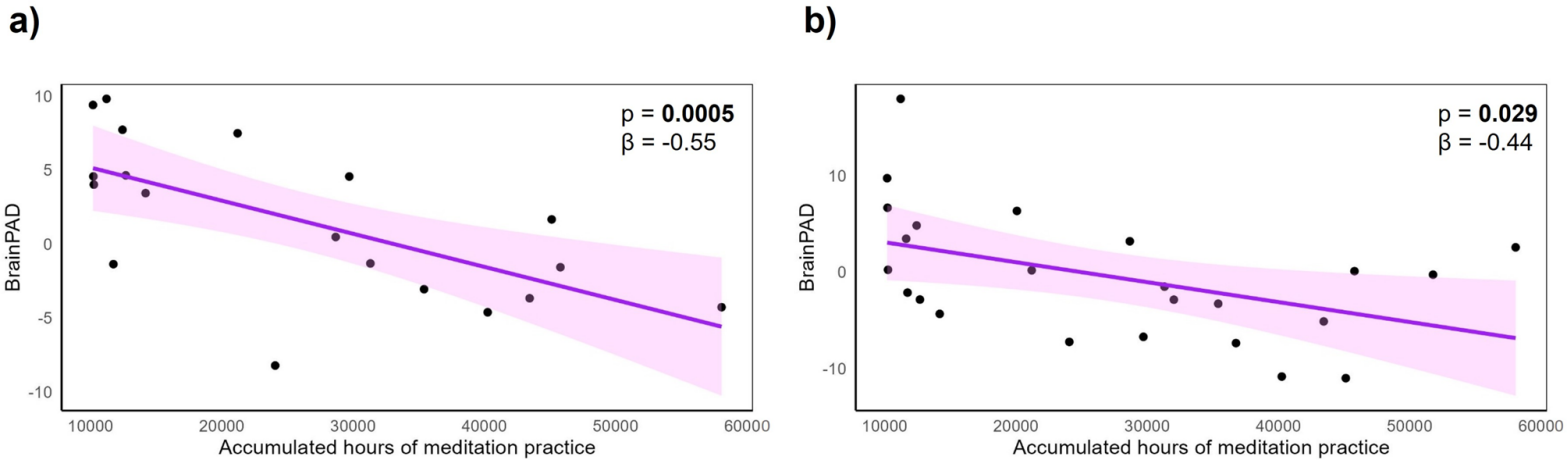
Associations between BrainPAD of OldExpMed and their accumulated hours of meditation practice. The scatter plots show the relationship between the accumulated hours of meditation practice and BrainPAD (**a**) with the main model and (**b**) with the replication model. Solid lines represent estimated regression lines, shaded areas represent 95% CI and β represents the slope of the regression. The regression was corrected for age for the main model. BrainPAD, brain predicted age difference; OldExpMed, older expert meditators; CI, confidence interval.

BrainPAD was significantly associated with the accumulated hours of meditation practice throughout life in OldExpMed, showing reduced/more negative BrainPAD with greater accumulated hours of meditation practice correcting for age (*p* = **0.0005** and β = − 0.55) (Fig. 2A).

### Links between BrainPAD and both cognitive and affective regulation capacity measures in OldExpMed

Results of the stepwise regression are reported in Table 2. As regard to cognitive performance, BrainPAD showed the strongest association with 2D-Mental Rotation Test time (2D-MRT, mental imagery: *p* = **0.008**, β = 0.45). For affective regulation capacities, BrainPAD showed the strongest link with the Prosocialness scale (*p* = **0.007**, β = − 0.75).

**Table 2:** Stepwise linear regressions showing the cognitive and affective regulation capacity measures most strongly associated with BrainPAD in OldExpMed.

	Coefficient [95% CI]	*p* value
Main model		
BrainPAD ~ 2D-MRT Time	0.6 [0.13, 1.01]	**0.008**
BrainPAD ~ Prosocialness score	− 0.36 [− 0.58; − 0.14]	**0.007**
Replication model		
BrainPAD ~ 2D-MRT Time	0.59 [0.21; 0.98]	**0.005**

BrainPAD, brain predicted age difference, OldExpMed, older expert meditators, CI, confidence interval, 2D-MRT, 2D-mental rotation test (for mental imagery). Significant values are in [bold].

### Longitudinal Impact of the 18-month meditation training in the CUOA

**Fig. 3:**
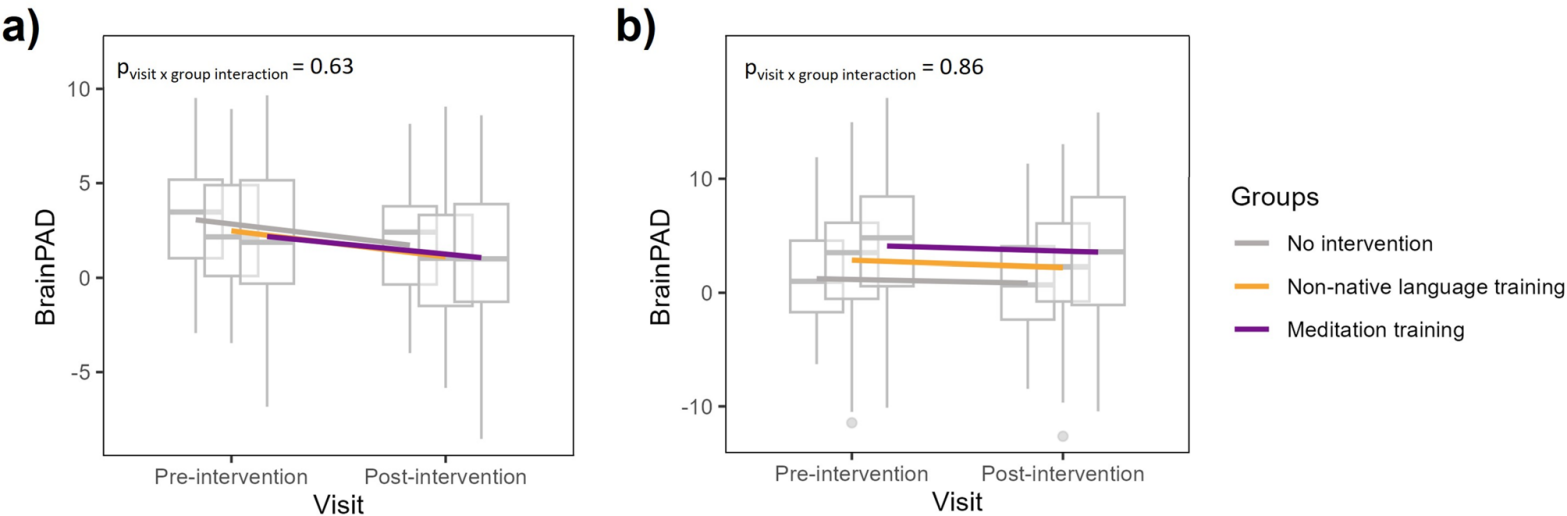
Evolution of BrainPAD from pre- to post-intervention in the three groups. Linear mixed models show no significant effects of the visit nor significant group x visit interaction for BrainPAD while controlling for age, sex and education. The boxplots show longitudinal change in BrainPAD (**a**) with the main model and (**b**) with the replication model. BrainPAD, brain predicted age difference*.*

The linear mixed model revealed no within-group changes nor significant between-group differences on BrainPAD (F_interaction_ = 0.46, p_interaction_ = 0.63) meaning that the meditation training showed no significant effect on BrainPAD within the group during the intervention nor compared to the other groups (Table 3 and Fig. 3A). Moreover, no significant association was found between the training responsiveness and BrainPAD in the meditation training group (*p* = 0.23, β = 0.22).

**Table 3:** Statistics of the within-group changes and between-group differences in BrainPAD from the linear mixed models.

	Main model				Replication model			
	N (baseline)	N (follow-up)	Estimate (95% CI)	*p* value	N (baseline)	N (follow-up)	Estimate (95% CI)	*p* value
Within-group standardized estimated change								
Meditation training	30	30	− 0.28 (− 0.5; 0.35)	0.18	45	45	− 0.07 (− 0.86; 0.72)	0.86
Non-native language training	30	30	− 0.07 (− 0.7; 0.14)	0.73	45	45	− 0.17 (− 0.96; 0.62)	0.68
No intervention	32	32	− 0.07 (− 0.48; 0.34)	0.73	44	44	0.08 (− 0.72; 0.87)	0.85
Mean difference in change between groups								
Meditation versus Non-native language training			− 0.21 (− 0.70; 0.28)	0.41			0.04 (− 1.05; 0.76)	0.83
Meditation training versus No intervention			− 0.21 (− 0.71; 0.29)	0.40			− 0.07 (− 0.80; 0.99)	0.75
Non-native language training versus No intervention			− 0.01 (− 0.49; 0.49)	0.99			− 0.13 (− 1.15; 0.66)	0.59

All analyses were adjusted for age, sex, and education. For within-group standardized estimated changes, positive values reflect increased BrainPAD within a trial group from baseline (pre-intervention) to post-intervention; negative coefficients indicate the opposite. For between-group differences, positive values reflect a relatively increased BrainPAD in a trial group (from baseline [pre-intervention] to post-intervention) compared to the specified reference group; negative coefficients indicate the opposite. Values are expressed as marginal means (95% CI). All *p* values were adjusted for multiple comparisons with Tukey correction. CI, confidence interval, BrainPAD, brain predicted age difference.

### Identification of the brain regions subtending brain age prediction

**Fig. 4:**
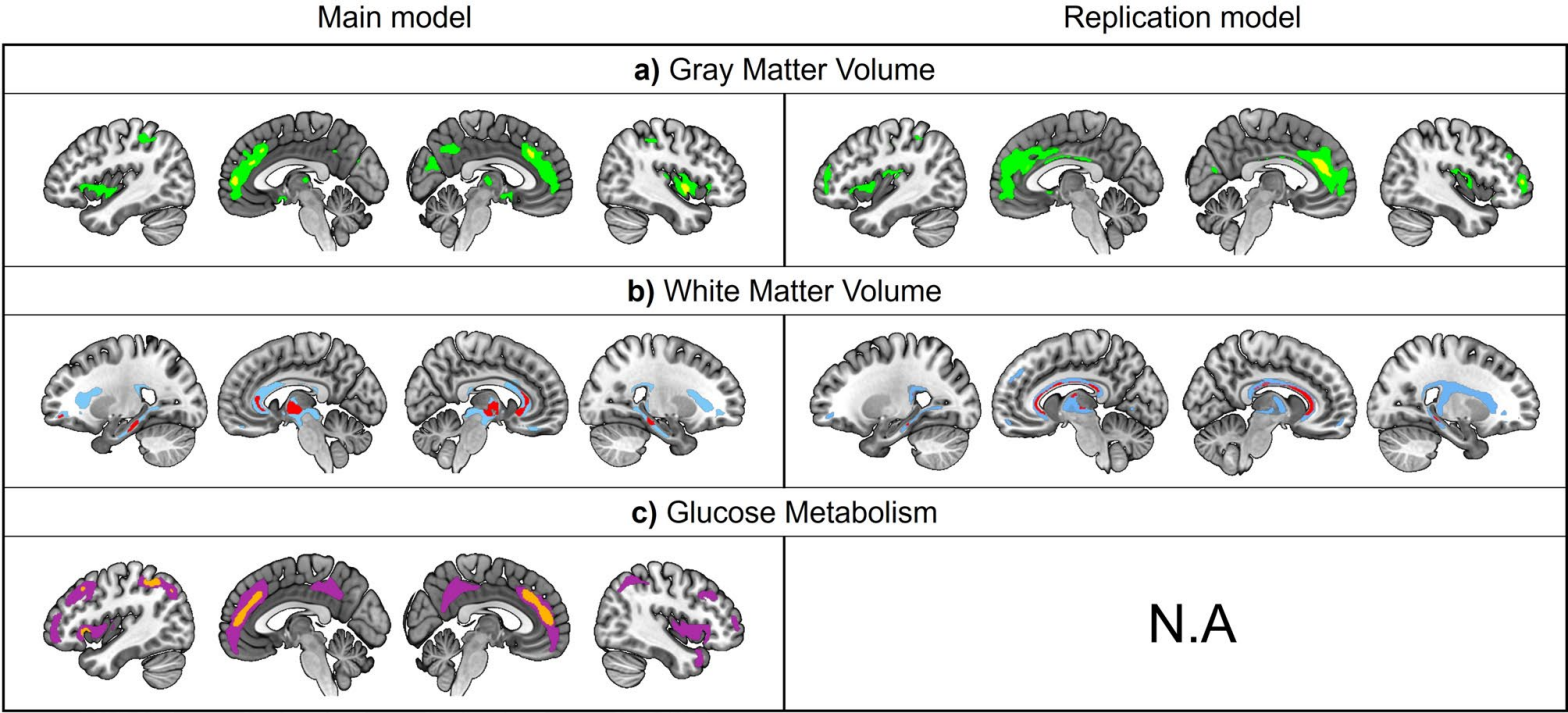
Voxel-wise associations between the predicted brain age and the different neuroimaging modalities used for brain age prediction. The results are projected on medial and external 3D brain surface views of the MNI template. (**a**) Associations between GMV maps and the predicted brain age at a threshold of *p* < 0.001 (green) and at *p*(FWE) < 0.05 (yellow). (**b**) Associations between WMV maps and the predicted brain age at a threshold of *p* < 0.001 (blue) and at *p* (FWE) < 0.05 (red). (**c**) Associations between glucose metabolism maps and the predicted brain age at *p* < 0.001(purple) and at *p*(FWE) < 0.05 (orange). MNI, montreal neurological institute; GMV, gray matter volume, FEW, family-wise errors; WMV, white matter volume.

For GMV, the predicted brain age was negatively associated with voxels in the frontal, temporal and parietal lobes including the insula, anterior cingulate cortex and orbitofrontal cortex. For WMV, the predicted brain age was negatively linked to voxels in frontal and temporal tracts such as the anterior corpus callosum, the hippocampal cingulum, and corona radiata. For glucose metabolism, BrainPAD negatively correlated with voxels in frontal, temporal and parietal regions, notably in the medial prefrontal cortex, insula and angular gyrus (Fig. 4).

### Results of the replication model

For replication, we used a previously developed model trained on multi-cohort Magnetic Resonance Imaging (MRI) data (GMV and WMV maps) from 2271 cognitively unimpaired individuals spanning the entire adult lifespan (age range: 30–97.2) using Gaussian Process Regression. The performance indices of the replication model were similar to those of the main model (predicted-chronological age correlation: *p*_test_ < 0.001, β = 0.47, MAE_test_ = 5.03, Supplementary Fig. S1B), although the MAE was slightly higher, indicating marginally inferior performance. Links with covariates were found to be similar, except for the correlation between age and BrainPAD which was not significant with the replication model (sex: *p* = 0.93, education: *p* = 0.98, age: *p* = 0.18). As a result, no demographic measures were included as covariates in the analyses in the OldExpMed group using the replication model. In the meditation training group, links with covariates were found to be similar, except for the correlation between education and ΔBrainPAD which was significant with the replication model (age: *p* = 0.27, β = 0.05; sex: *p* = 0.83, β = 0.07; education: *p* = **0.03**, β = 0.18).

For the difference between OldExpMed and meditation-naive CUOA, the replication model yielded similar results, i.e. OldExpMed had a significantly lower/more negative BrainPAD compared with CUOA (F = 8.15, *p* = 0.004, Fig. 1B). For the links between BrainPAD and OldExpMed accumulated hours of practice, a significant association was also found with the replication model (*p* = 0.029, β = − 0.44, Fig. 2B). For the association with cognitive measures, the best predictor of BrainPAD was also the 2D-MRT time (mental imagery: *p* = 0.005, β = 0.59) for cognition. However, for the affective regulation capacities, no variable entered the model (Table 2). For the longitudinal analysis of the effect of the 18-month meditation training in the CUOA, the results were identical to those with the main model, showing no within-group changes nor significant between-group differences on BrainPAD (Fig. 3B and Table 3) nor any association, in the meditation training group, between ΔBrainPAD and the training-responsiveness score (*p* = 0.92, β = − 0.02). Finally, for the regions subtending brain aging estimation, the predicted brain age was negatively associated with voxels in frontal, temporal and parietal regions for GMV, notably in the insula, medial prefrontal cortex, and posterior cingulate cortex, while it was negatively linked to WMV in frontal and temporal tracts such as the corpus callosum and the hippocampal cingulum. However, no result survived at a threshold of p (Family-Wise Errors [FWE]) < 0.05 (Fig. 4).

## Discussion

Our study aimed to assess the impact of long-term meditation expertise and an 18-month meditation training on brain age, estimated with machine learning based on multimodal neuroimaging. We found that, at the same chronological age, OldExpMed had a younger estimated brain age compared to meditation-naive CUOA. Additionally, this effect was stronger with more accumulated practice hours and was linked to better cognitive and affective regulation capacity scores. However, contrary to our hypothesis, the 18-month meditation training showed no effect on brain aging.

We found a significant difference in BrainPAD between OldExpMed and CUOA, with OldExpMed presenting younger brains, suggesting reduced brain aging, and this result was consistent across both the main and replication models. From a global perspective, our findings align with the existing literature, which consistently reports greater structural brain preservation in expert meditators compared to non-meditators^15–17,31–33^. Specifically, previous studies have demonstrated greater cortical volume in older expert meditators compared to controls^15–17^, as well as a less steep slope in the cross-sectional correlation between GMV and age in expert meditators, suggesting reduced age-related GM decline compared to non-meditators^31–33^. Our findings also align with the only two previous studies using a similar machine learning approach to assess brain age in expert meditators – one involving a single expert and the other 50 expert meditators aged 24–77 years^30^ – both of which reported lower/more negative BrainPAD compared to age-matched controls.

Additionally, in OldExpMed, we highlighted an association between brain aging and the accumulated hours of meditation practice throughout life, with more hours of practice linked to a younger brain. This suggests a potential causal role of the meditation experience in the slowed brain aging observed in OldExpMed, further highlighting its promise as a tool for promoting healthy aging – although confirmation would be needed especially as we found no effect in our longitudinal study (see below). Moreover, unaccounted factors such as possible lifestyle differences between OldExpMed participants and meditation-naive CUOA (e.g., variation in diet, alcohol and tobacco consumption, or stress levels) may have contributed to the observed effects. As such, the slowed brain aging may result from a combination of meditation expertise and the distinctive lifestyle often associated with long-term meditation practice.

We also found that slower brain aging in OldExpMed was associated with greater performance in mental imagery, both in the main and in the replication models, suggesting that the meditation-related slower brain aging translates into, and/or is mediated by, better maintenance of mental imagery capacity in these individuals. This association may reflect meditation’s role in preserving or enhancing neuroplasticity and connectivity in brain regions critical for visuospatial processing^34–39^ and cognitive control^37,39–41^, which support complex visualization tasks^34,42,43^. Meditation practice, which often involves attention and imagery exercises, might contribute to maintaining the neural efficiency and integrity necessary for these cognitive abilities, counteracting typical age-related decline. Interestingly, mental imagery has been shown to play a significant role in various mental and neurological disorders, such as anxiety, which suggests that it may have a beneficial impact in their treatment^44^. Given the association between BrainPAD and mental imagery in OldExpMed, greater mental imagery may serve as a mechanism through which meditation promotes healthy aging, potentially by reducing anxiety.

In addition, we observed that a younger brain in OldExpMed was associated with higher prosocialness scores with our main model. While the relationship between meditation and prosocial behavior is well established^45–48^, most evidence comes from interventional studies and not specifically in older adults. Two meta-analyses have shown moderate increase in prosociality following a meditation intervention^46,48^. However, neuroimaging studies have also shown that increased metabolism or GMV in regions associated with increased prosocial behavior, such as the anterior cingulate cortex, insula, and orbitofrontal cortex^49,50^, are also enhanced in older expert meditators^16,39,51^. Given these connections, it is not surprising that we found a relationship between BrainPAD and prosocialness in OldExpMed. Our findings suggest that long-term meditation practice in older adults may be linked to structural brain characteristics that support prosocial tendencies, which are likely cultivated and reinforced over decades of practice. This association could reflect the impact of sustained meditation on promoting emotional regulation and empathy, both of which are key components of prosocial behavior^46,47,52^ and are known to be influenced by meditation practice^53,54^. Further studies are needed to confirm and expand upon the cognitive and affective regulation capacity correlates of BrainPAD changes related to meditation expertise, especially given the limited sample size and the lack of replication in some findings when using alternative models.

In contrast to our findings in OldExpMed, and contrary to our hypothesis, we did not observe a significant effect of the 18-month meditation training on brain aging. The meditation training group showed no evidence of slowed brain aging, either within the group during the intervention or when compared to the other groups (non-native language and no intervention groups). Additionally, we found no association between BrainPAD changes and the training-responsiveness score within the meditation training group. While we did not observe an effect of the meditation training on brain volume and perfusion in the two key regions selected as our main outcomes^20^, we anticipated that a more integrative and exhaustive technique, such as the machine learning approach used here, might detect more subtle differences and meaningfully integrate several sources of information, potentially reflected in a younger brain age. However, the structural MRI and FDG-PET modalities used here may not capture functional aspects of brain activity or connectivity patterns. Including functional MRI in future studies may help detect subtle neural changes induced by meditation that are not reflected in anatomy or metabolism. This lack of significant results may be attributed to our participants’ high education levels and healthy lifestyles, which are associated with high cognitive and brain reserve, potentially limiting the detectable effects of meditation. Another possibility is that, despite generally high adherence, individual differences in engagement with the intervention may have diluted potential effects. The fact that we found an association between BrainPAD and the accumulated hours of meditation practice in OldExpMed, which ranged from 19 to 44 years, suggests that the 18 months of training in the longitudinal study may have been insufficient to produce noticeable effects on brain aging. Alternatively, although the correlation between Brain Age and the accumulated hours of meditation practice in OldExpMed suggests a link, it is still possible that observed effects may be attributed to other baseline differences or lifestyle factors, as the cross-sectional design limits causal inferences. Future studies should investigate whether the meditation training itself leads to a younger brain age and determine the minimum number of hours of practice needed to achieve a significant impact on brain aging.

Finally, we aimed to identify the brain regions underlying the brain age predictions made by our models. We found that slower brain aging correlates with increased GMV and glucose metabolism in frontal, temporal and parietal regions including the insula, anterior cingulate cortex, orbitofrontal cortex and angular gyrus. These regions have consistently shown greater preservation in expert meditators compared to meditation-naive controls^16,17^ and are commonly reported in studies examining the effects of meditation^55^. Furthermore, these areas are particularly sensitive to aging^56,57^ suggesting that our model successfully captured differences in brain aging between OldExpMed and meditation-naive CUOA by estimating brain age based on variations in these relevant regions. Similarly, correlations with WMV with the main model also highlighted a tract commonly found in studies investigating the links between meditation and WM: the corpus callosum^55^. Notably, similar regions were associated with BrainPAD with the replication model as with the main model. This suggests that, in accordance to our hypothesis, the differences observed in the results between the two models might have resulted from the addition of FDG-PET data which may lead to a more precise prediction of brain aging by relying on a modality particularly sensitive to early brain changes. Indeed, we observed that the main model has a lower MAE – which indicates a greater precision in the prediction – as well as lower *p* value for most analyses.

To strengthen interpretability, we employed two complementary brain age prediction models that differed in training samples, imaging modalities, and algorithms. The main model, tailored for this study, was trained on older adults using structural (GMV, WMV) and functional (FDG-PET) data and Lasso regression, optimizing its sensitivity to late-life brain changes. In contrast, the replication model was a pre-existing lifespan model trained on a broader age range using structural MRI and Gaussian Process Regression. Subtle variations in statistical outcomes across models reflect their respective design sensitivities—lifespan generalization versus age-specific precision and functional integration. Importantly, convergence across models in key results supports the robustness of our conclusions.

After adjusting for age, the difference in BrainPAD between OldExpMed and CUOA was no longer significant in the main model, whereas it remained significant in the replication model. In the main model, the loss of significance could reflect a genuine but modest group difference that becomes harder to detect when statistical power is reduced and collinearity is increased by including a covariate closely related to the outcome. It may also be influenced by the smaller number of training subjects in this model, leading to less stable estimates. Alternatively, the original effect might have been partially driven by residual age-related bias; however, the persistence of significance in the replication model argues against this being the sole explanation. Given the ongoing debate regarding age adjustment in BrainPAD analyses and the potential for residual age bias, future studies should ideally report results both with and without age correction.

This study has several strengths and limitations. A key strength lies in the integration of multiple sources of information to estimate brain integrity through brain age, providing a more accurate measure of brain health. The inclusion of a second model enabled us to replicate our main findings, enhancing the robustness of the results. Numerous brain age models exist, and the goal of this study was not to conduct exhaustive tests of these models or to identify the best predictive ones. Instead, we aimed to assess whether meditation influences brain age, and to achieve this we tested two complementary models, each with its respective advantages and disadvantages, to provide a robust confirmation of our findings. In addition, by examining both the long-term effect of meditation expertise over decades and the shorter term effect of an 18-month meditation training (interventional setting), we were able to comprehensively investigate the effects of meditation on brain aging. However, some limitations should be acknowledged. The cross-sectional nature of our analyses on OldExpMed precludes causal inferences about the effects of long-term meditation; longitudinal studies with longer meditation training program are needed to confirm these findings. Our control group was not fully representative of the general population, being highly educated and active, which may have limited the ability to detect effects in the longitudinal analyses. Moreover, although our sample of OldExpMed was relatively large compared to similar studies focusing in older expert meditators, it remains small, limiting the statistical power to observe more robust effects. Additionally, the inclusion of age as a covariate in BrainPAD analyses remains a point of methodological discussion, and our findings should be interpreted in light of this consideration. Finally, while we applied the same preprocessing pipeline and selected cognitively healthy older adults from the ADNI dataset to match the Age-Well sample, differences in scanner types and acquisition protocols between datasets may have introduced variability and limited generalizability. Although we attempted to mitigate these effects through preprocessing and feature standardization, residual site-related variance may persist, and we now explicitly acknowledge this limitation.

In conclusion, we found that OldExpMed exhibited slower brain aging compared to meditation-naive CUOA, with this effect becoming more pronounced with longer meditation practice. Additionally, this slower brain aging was associated with greater cognitive and affective regulation capacity measures. In contrast, an 18-month meditation training did not yield any observable effects on brain aging in older participants. Together, these findings underscore the potential role of meditation in promoting healthy aging and highlight the need for future research to confirm the causal relationship between meditation and brain aging, as well as to determine the necessary meditation dose required to observe this effect.

## Material & methods

### Participants of the cross-sectional study

The design and method of the Age-Well observational study on expert meditators and the Age-Well trial protocol have been described previously^58,59^. We included data from 27 OldExpMed as well as 137 CUOA with no previous experience in meditation, all from the Age-Well Randomized Clinical Trial (RCT) of the Medit-Ageing European project^58,59^. OldExpMed were recruited in Europe through flyers, advertisements in Buddhist magazines, emails, and presentations in Buddhist meditation retreat centers. OldExpMed had extensive practices in two families of Buddhist meditations: mindfulness meditation and loving-kindness and compassion meditation; having practiced both families of meditation for at least 10 000 h in their lifetime. Their practice had to be regular (i.e., at least 6 days a week for at least 45 min) and include at least 6 cumulative months spent in retreat (i.e., practicing meditation for at least 8 h a day). From the initial sample of 27 OldExpMed, 7 participants were excluded from analyses derived from FDG-PET data as they did not have available scans. Additionally, two of these participants were excluded from analysis derived from MRI due to a change of MRI machine (c.f. flowchart in Supplementary Fig. S2,S3 for details). The CUOA were recruited from the general population, were all native French speakers, aged over 65, educated for at least 7 years and cognitively unimpaired. They had no evidence of major neurological or psychological disorders, no history of cerebrovascular disease, no chronic disease, or acute unstable illness and no current medication that may interfere with cognitive functioning. Out of the 137 CUOA, two were excluded from the analyses: one was diagnosed with amyotrophic lateral sclerosis, and one had a past experience of a head trauma with loss of consciousness for more than one hour. Participants meeting inclusion criteria underwent a detailed neuropsychological assessment, blood tests, structural MRI and FDG-PET scans. All participants gave their written informed consent prior to the examinations.

### Design of the longitudinal study

Age-Well was a monocentric, randomized, observer-masked, controlled superiority clinical trial with three parallel arms: an 18-month meditation training arm, an 18-month non-native language (English) training arm and a no intervention arm. Participants underwent multimodal assessments including cognitive, emotional, affective, neuroimaging, and biological assessments. Data reported here corresponded to post-intervention versus pre-intervention (baseline). Expert meditators, and experienced English teachers, were involved in defining selection criteria, shaping the meditation or non-native language training respectively, and reviewing study materials. Age-Well was carried out in Caen (France). All experimental protocols [Age-Well RCT] were approved by local ethics committee (Comité de Protection des Personnes CPP Nord-Ouest III, Caen; trial registration number: EudraCT: 2016–002,441-36; IDRCB: 2016-A01767-44; ClinicalTrials.gov Identifier: NCT02977819, registration date: 30/11/2016). The study was conducted in accordance with the Declaration of Helsinki.

### Participants of the longitudinal study

Participants were enrolled between November 24, 2016, and March 5, 2018. We included baseline and longitudinal data from 137 CUOA from the Age-Well RCT of the Medit-Ageing European Project^59^, sponsored by the French National Institute of Health and Medical Research (INSERM). The Age-Well trial protocol has been described in previous publications^59^, and is further detailed in the Supplementary materials (Supplementary methods 2.1). From the initial sample of 137 participants, two were excluded from the trials after randomization for previously listed reasons. In addition, another participant died before the end of the trial (myocardial infarction, not related to the study) and one participant revealed not to have followed the allocated arm (randomised to no intervention but attended non-native language training and was analysed within the non-native language training arm). The flowchart of the longitudinal study is represented in Supplementary materials (Supplementary Fig. S4). The main inclusion criteria were being over 65 years old, having completed at least 7 years of education, performing within the normal range for age and educational level on standardized cognitive tests, and having no history of major neurological or psychiatric disorders. Additionally, participants should not have had a strong preference or aversion to any intervention group, no current or past regular or intensive practice of meditation or comparable practices and should not have been fluent in spoken English. Participants meeting inclusion criteria underwent a detailed neuropsychological assessment, structural MRI and FDG-PET scans. All participants gave their written informed consent prior to the examinations. Adverse events and serious adverse events were recorded throughout the study when reported by the participants and systematically at each study visit during a consultation with a physician.

### Randomization and blinding

Eligible participants were randomized after their baseline assessment. They were randomly assigned (1:1:1) to the meditation training, non-native language training or no-intervention arm according to a randomization list with permuted blocks of varying size (6 and 9), which was generated centrally by a biostatistician at the European Clinical Trials Platform & Development group (Euclid, Bordeaux, France) prior to the start of the study. Individual allocation results were concealed in sealed envelopes. All study personnel, including the investigators and outcome assessors, were masked to treatment allocation. Only the intervention facilitators, trial-independent statisticians, and data monitoring infrastructure staff were unmasked or partially unmasked.

### Interventions

The meditation and non-native language training interventions were structurally equivalent in overall course length, class time, and home activities, and were matched in administration, dosage, duration, and level of expertise and number of facilitators per class. For both the meditation training and the non-native language training groups, interventions were 18-month in length and comprised 2-h weekly group sessions and one day of more intense practice (5 h during the day).

#### Meditation training

The meditation training consisted of an original secular program of meditation training labelled “The Silver Santé Study Meditation Program”, specifically designed for this study based on pre-existing interventions with the objective of personal development and healthy aging, and was provided by expert meditator instructors in Caen. The objective of this 18-month intervention program was to develop mindfulness, kindness, and compassion abilities as additional psychological resources to cope with challenges related to aging on physical, cognitive and psychological aspects^59,60^. Each session contained moments of group meditation, sitting or walking and moments of sharing and teaching. Full details can be found in the Supplementary materials (Supplementary methods 2.1.2) and in the protocol paper of the Age-Well study^59^.

#### Non-native language training

The non-native language training program was a cognitively stimulating program. It consisted of English exercises designed to reinforce each participant’s abilities in understanding, writing, and speaking. Sessions were held by mixing oral comprehension and expression activities to prioritize the acquisition of new vocabulary and grammatical structures. Full details can be found in the Supplementary materials (Supplementary methods 2.1.3) and in the protocol paper of the Age-Well study^59^.

#### No intervention group

Participants in the no intervention group were requested not to change their habits and to continue living as they did before engaging in the study and until its conclusion. They were specifically asked not to engage in meditation or non-native language training during the 18-month period of the study^59^.

### Neuroimaging assessment

All participants included in the analyses were scanned on the same MRI (Philips Achieva; 3.0 T) and PET cameras (Discovery RX VCT 64 PET-CT; General Electric Healthcare) at the Cyceron Center (Caen, France).

#### MRI data acquisition and pre-processing

High-resolution T1-weighted structural imaging was acquired to measure GMV using a 3D fast-field echo sequence (3D-T1-FFE sagittal, repetition time = 7.1 ms, echo time = 3.3 ms, flip angle = 6°, 180 slices with no gap, slice thickness = 1 mm, field of view = 256 × 256 mm^2^, in-plane resolution = 1×1x1 mm^3^). T1-weighted images were first segmented using multimodal segmentation (with Fluid Attenuated Inversion Recovery images) (https://www.fil.ion.ucl.ac.uk/spm/docs/tutorials/vbmcourse24/preprocessing/), then spatially normalized to the Montreal Neurological Institute (MNI) template, and modulated for nonlinear warping (to correct for non-linear warping so that values in resultant images are expressed as volume corrected for brain size) using the Segment function of the Statistical Parametric Mapping software version 12. The resulting local GMV and WMV maps corrected for brain size were used for brain age prediction.

#### PET data acquisition and pre-processing

FDG-PET scans were acquired with a resolution of 3.76 × 3.76 × 4.9 mm^3^ (FOV 157 mm). Forty-seven planes with a voxel size of 1.95 × 1.95 × 3.27 mm^3^ were obtained. Before the PET acquisition, a transmission scan was performed for attenuation correction. Participants (n = 92) fasted for at least 6 h before scanning. After a 30-min resting period in a quiet and dark environment, ∼180 MBq of FDG was injected intravenously as a bolus and a 10-min PET acquisition scan was acquired 50 min after injection. PET images were coregistered on their corresponding T1-weighted MRI, and were then normalized to the MNI template using deformation parameters derived from the anatomical MRI. The resulting images were quantitatively normalized using the cerebellum. The resulting PET images were used for brain age prediction.

### Neuropsychological assessment

Participants completed a series of neuropsychological tests and questionnaires, which provided insight into various aspects of their cognition (global cognition, attention, executive functions, working memory, episodic memory…), meditation-related traits (mindfulness, interoception, compassion), emotions (anxiety, depression, emotion regulation, worry, rumination), well-being, prosociality and loneliness. Full details on the specific tests and questionnaires used, along with their descriptions, are provided in the Supplementary materials (see Supplementary methods 2.2).

### Training-responsiveness composite score

In both intervention groups (meditation and non-native language groups), we assessed whether and to what degree participants responded to the interventions by a continuous measure of responsiveness that evaluated the acquired skills at the end of the interventions, using different scales for each training to compute these scores^61^.

### Brain age estimation methods

**Fig. 5:**
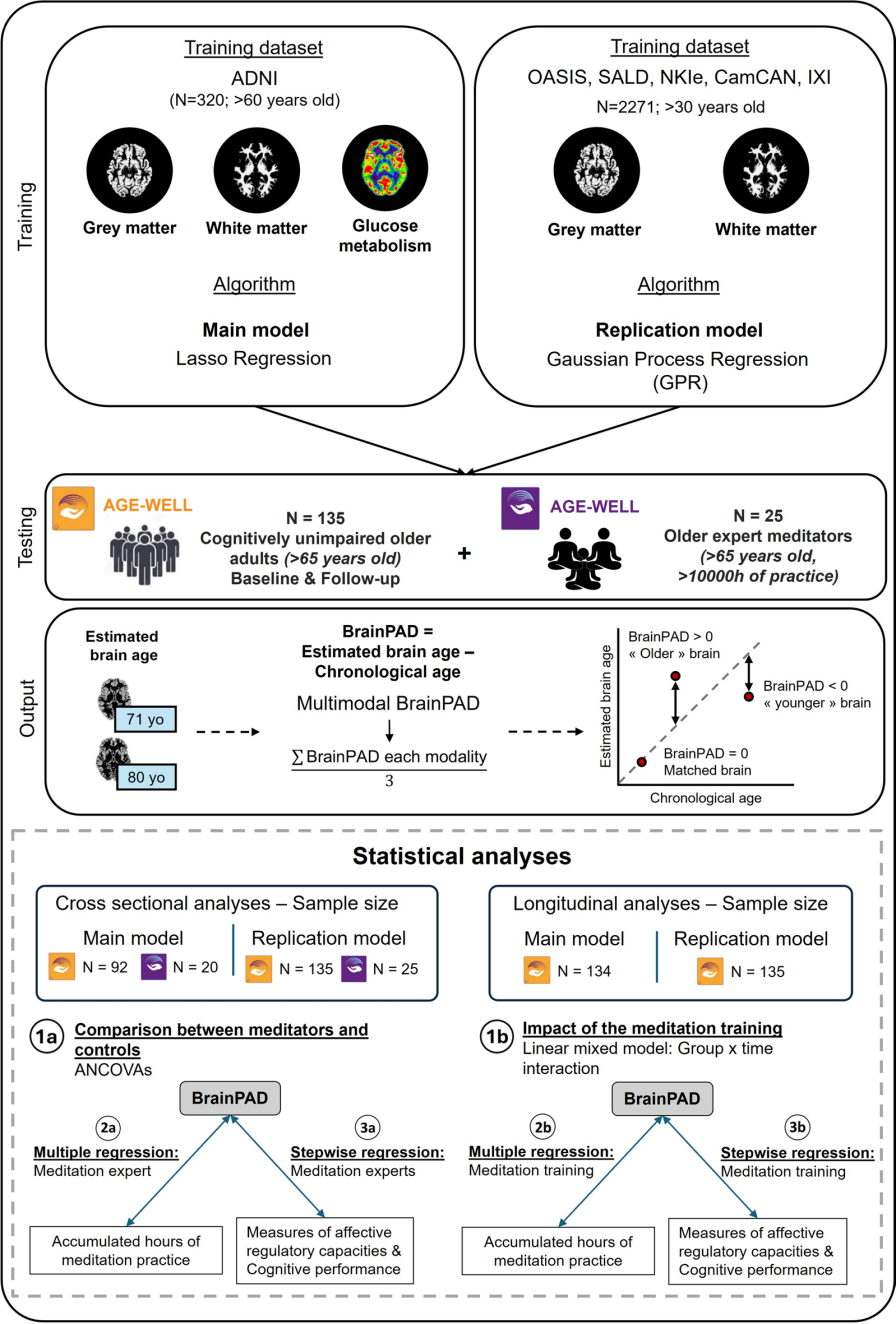
Design overview**.** Two models were used for Brain age prediction: the main model, trained using both structural MRI (GMV and WMV) and functional FDG-PET data from cognitively unimpaired elderly individual from ADNI with Lasso regression; and the replication model, trained on a larger, multi-cohort dataset spanning the entire adult lifespan using only structural MRI data (GMV and WMV) and applying Gaussian Process regression. Both trained models were applied to Age-Well participants, divided into two groups: CUOA (N = 135, age > 65 years) and OldExpMed (N = 25, age > 65 years, > 10,000 h of practice). As output, the models yielded the estimated brain age which was used to calculate BrainPAD by subtracting the chronological age from the predicted brain age for each modality. A multimodal BrainPAD score was derived by averaging BrainPAD values across the available imaging modalities. Bottom panel: First, using a cross-sectional design, a comparison between OldExpMed and CUOA at baseline was performed (**1a**). Then, if the difference was found significant, we investigated the links—in OldExpMed—between BrainPAD and the experts’ accumulated duration of practice (**2a**), cognitive performance, and affective regulation capacities (**3a**). Second, using a longitudinal design, we investigated the impact of the meditation training on BrainPAD in the cognitively unimpaired older adults using a linear mixed model (**1b**). If the interaction between group and time was found significant, we investigated—in the meditation training group—the links between ΔBrainPAD and the accumulated hours of practice (**2b**), cognitive performance, and affective regulation capacities (**3b**). MRI, magnetic resonance imaging, GMV, gray matter volume; WMV, white matter volume; FDG-PET, fluorodesoxyglucose-positron emission tomography; ADNI, Alzheimer’s disease neuroimaging initiative, CUOA, cognitively unimpaired older adults, OldExpMed, older expert meditators; BrainPAD, brain predicted age difference, ΔBrainPAD, longitudinal evolution of BrainPAD.

We used two models for Brain age prediction (Fig. 5). The first one (the main model), described in detail below, was trained by us using both structural MRI (GMV and WMV) and functional FDG-PET data from cognitively unimpaired elderly individuals, applying Lasso regression. In contrast, the second (replication) model, detailed in the Supplementary materials (Supplementary methods 2.3), was a previously established one^62^ trained on a larger, multi-cohort dataset spanning the entire adult lifespan using only structural MRI data (GMV and WMV) and applying Gaussian Process regression. The main model was expected to be particularly sensitive and specific in our test population for several reasons. First, it was trained on a sample of older adults, thereby increasing its relevance for aging-related prediction. Second, it incorporated multiple imaging modalities, including FDG-PET, which is known to be more sensitive than structural MRI in detecting early metabolic changes in aging. Importantly, prior studies have shown that combining imaging modalities significantly enhances brain age prediction accuracy compared to single-modality models^63,64^. These studies found that multimodal models yielded lower prediction error. The second model, trained on a broader sample across the full adult age range, serves as a robust replication tool^62^. Its larger and more diverse training dataset enhances its reliability and stability, providing complementary validation of our findings. While it may be less specific for older adults due to its broader focus, using both models together strengthens the overall approach. Their complementarity allows us to validate our results from two perspectives: one highly sensitive and specific to elderly populations, and the other more generalizable across a wider age range – thus ensuring greater robustness and confidence in our conclusions.

To train the main model, we used GMV, WMV, and FDG-PET maps of 320 healthy subjects from the ADNI dataset (age range: 60–96 years old, mean = 75.9 years old, standard deviation [SD] = 6.5 years old) (Fig. 5). Details regarding the cohort recruitment and data collection methods are available online (http://adni.loni.usc.edu/). The acquisition processes for structural MRI imaging are described at http://adni.loni.usc.edu/data-samples/data-types/. The model was trained using Lasso regression, a method designed to address the problems of overfitting and multicollinearity in ordinary least square regression^65^. It imposes an L1-norm penalty, in which the goal is to minimize the absolute values of the beta coefficients in the model. This approach allows the model to select informative features and discard uninformative ones^66^. Lasso was selected based on prior comparative studies in the brain age prediction literature, where it was found to perform equally well or better than other machine learning algorithms such as Support Vector Regression, Relevance Vector Regression, or Random Forests^65,67,68^, and on our own empirical testing, where Lasso yielded the best performance across several training datasets and combinations of imaging modalities. This model was developed using content from the scikit-learn library (https://scikit-learn.org/stable/modules/generated/sklearn.linear_model.Lasso.html). The optimal value of the regularization parameter (Lambda) was determined using 20-fold cross-validation. After training the model, it was then applied to the Age-Well images for each modality (T1-weighted GMV and WMV maps and FDG-PET) to obtain one predicted brain age for each imaging modality.

BrainPAD^69,70^ was then computed for each participant by subtracting their chronological age from the predicted brain age, for each modality. The resulting differences were then averaged to produce a single indicator of brain aging for each participant. A positive BrainPAD (predicted age > chronological age) indicates accelerated brain aging while a negative BrainPAD (predicted age < chronological age) indicates slower brain aging (Fig. 5).

### Sample size

Age-Well was powered to detect an effect size of 0.75 for the trial’s coprimary outcomes (i.e. volume and perfusion of the anterior cingulate cortex and insula), with 80% power and a 2-sided type I error of 1.25%^20^. This resulted in a minimum of 126 participants (42 per arm), which was exceeded (137 total participants). Following guidance^71^, post-hoc power analyses were not performed for this secondary outcome study.

### Statistical analyses

#### Accuracy of the model

First, we evaluated the accuracy of the model by performing a correlation in the training sample between the predicted cerebral age and the chronological age as well as by calculating the MAE. The MAE is a commonly used metric to evaluate the accuracy of brain age predictions. These metrics provide indicators of model performance: a high correlation reflects strong alignment between predicted and actual ages, and lower MAE values indicate greater prediction accuracy and lower deviation from true ages. For the MAE, values below 5 years are generally considered good^65^, indicating that, on average, the model’s predictions are within 5 years of the true age. Previous studies have shown that the MAE typically ranges from 4 to 8 years when using structural features^27,65,72^.

#### Covariate assessment

To assess how the most relevant covariates (age, sex and level of education) might interfere with our findings, we first assessed whether they differed between OldExpMed and meditation-naive CUOA (two sample t-tests). Second, we performed linear regressions to assess the links between BrainPAD and these covariates (main effect of each covariate) and examined whether these associations differed between groups (interaction effects between each covariate and the group).

#### Cross-sectional analyses of OldExpMed

First, to compare BrainPAD between OldExpMed and meditation-naive CUOA (Fig. 5, 1a), we conducted analyses of covariance. We included demographic variables (age, sex, and education) as covariates if they were significantly different between OldExpMed and CUOA or if their relationship with BrainPAD varied significantly between the groups (i.e., significant interaction effects).

Second, if the difference between OldExpMed and CUOA was found significant, we investigated the links – in OldExpMed – between BrainPAD and OldExpMed accumulated hours of practice throughout life (Fig. 5, 2a) as well as their cognitive performance and affective regulation capacities (Fig. 5, 3a). The links between BrainPAD and the accumulated hours of meditation practice were assessed with a linear regression, excluding the outliers, i.e. OldExpMed with more than ± 3 SD from the group mean of accumulated hours of meditation practice (n = 1, see Supplementary materials, Supplementary Fig. S5. To identify the cognitive and affective regulation capacity measures most associated with BrainPAD, we employed stepwise regressions (backward then forward). Linear and stepwise regressions were performed in R Studio. Demographic variables were included as covariates in these analyses if their association with BrainPAD in the OldExpMed group was significant.

#### Longitudinal analyses of the effect of the 18-month meditation training in the CUOA

First, for the longitudinal assessment of the effects of the meditation training, we examined within-group changes and between-group differences to investigate the specific impact of the 18-month meditation training on BrainPAD in CUOA (Fig. 5, 1b). We used a linear mixed model including intervention groups (i.e., meditation, non-native language, and no intervention groups), visits (i.e., pre- and post-intervention), and an interaction between intervention groups and visits as fixed effects, and with participant-level random intercepts, estimated via restricted maximum likelihood. In accordance with the pre-registered statistical analysis plan for secondary outcomes of the Medit-Ageing Project, baseline age, sex, and education level were included as fixed effects, with continuous variables mean-centered. Tukey post-hoc tests were used to examine within- and between-groups changes. Linear mixed models were performed with the *lme4* and *emmeans* packages implemented in R Studio.

Second, if the interaction between intervention groups and visits was found significant, we investigated the links between ΔBrainPAD and the accumulated hours of practice (Fig. 5, 2b), cognitive performance, and affective regulation capacities of the CUOA participants (Fig. 5, 3b). Demographic variables (age, sex, and education) were included as covariates in these analyses if their association with BrainPAD in the CUOA group was significant.

Finally, regression between ΔBrainPAD and training responsiveness in the meditation training group was performed. Demographic variables (age, sex, and education) were included as covariates in these analyses if their association with ΔBrainPAD in the meditation training group was significant.

#### Identification of brain regions subtending brain age prediction

Finally, we assessed which brain regions were the most informative for the model to predict brain aging by exploring the association – in OldExpMed and CUOA – between the predicted brain age and the brain imaging modalities used for brain age prediction. To that end, we performed multiple regressions on both structural MRI (GMV and WMV maps) and FDG-PET data using a threshold of p (cluster-level uncorrected) < 0.001 with a value of k determined by Monte-Carlo simulation using the 3dClustSim program (Analysis of Functional NeuroImages 18.0.11) to achieve a corrected statistical significance of *p* < 0.05. Additionally, we also used a voxel-level threshold of *p* < 0.05 corrected for FWE to assess which regions showed the strongest association.

#### Replication model analyses

All statistical analyses were replicated using the alternative model for brain age prediction.

#### Adjustment for chronological age in BrainPAD analyses

The inclusion of chronological age as a covariate in BrainPAD analyses is a matter of debate. Arguments against such adjustment include that age is already mathematically embedded in the outcome (BrainPAD = predicted age – chronological age), so further adjustment may introduce over-adjustment or collinearity. Moreover, because our groups were age-matched, the rationale for additionally controlling for age is reduced^73^. Conversely, because BrainPAD often remains correlated with age (“age bias”), failing to adjust may allow this residual association to confound group comparisons or correlations with other variables^73,74^. In the sake of completeness, we present the main results without age as a covariate (consistent with our statistical analysis plan) and provide complementary analyses including age as a covariate in the Supplementary Materials.

## Supplementary Information

Supplementary Information.

## Data Availability

The data underlying this report are made available on request following a formal data sharing agreement and approval by the consortium and executive committee. The data sharing request form can be downloaded at https://silversantestudy.eu/2020/09/25/data-sharing/. Statistical code and other study materials are available upon reasonable request from Dr. S. Haudry. The ADNI dataset is publicly available at https://ida.loni.usc.edu/login.jsp?project=ADNI and IXI at https://brain-development.org/ixi-dataset/, Cam‐CAN is available under https://camcan-archive.mrc-cbu.cam.ac.uk/dataaccess/, OASIS‐3 can be found on https://sites.wustl.edu/oasisbrains/#data, Enhanced NKI‐RS dataset is available at https://fcon_1000.projects.nitrc.org/indi/pro/eNKI_RS_TRT/FrontPage.html, and the SALD dataset can be downloaded from https://fcon_1000.projects.nitrc.org/indi/retro/sald.html.
