# Supplementary material for: Impact of meditation on brain age derived from multimodal neuroimaging in experts and older adults from a randomized trial
Source: Sci Rep. 2025 Oct 28;15:37710. doi: 10.1038/s41598-025-21490-9 (PMC12569194; doi:10.1038/s41598-025-21490-9)
Supplement: Supplementary file 1 — Supplementary Information. [file 41598_2025_21490_MOESM1_ESM.docx]

**Supplementary for “Impact of Meditation on Brain Age Derived from Multimodal Neuroimaging in Experts and Older Adults from a Randomized Trial ”**

Sacha Haudry, Natacha Lambert, Christian Gaser, Bertrand Thirion, Brigitte Landeau, Julie Gonneaud, Géraldine Poisnel, Pierre Champetier, Asrar Lehodey, Natalie L. Marchant, Olga Klimecki, Fabienne Collette, Denis Vivien, Vincent de la Sayette, Antoine Lutz, Gaël Chételat

Table of contents

1. Supplementary results…………………………………………………………………………………………………………… 3

1.1. Replication model……………………………………………………………………………………………………………..3

1.1.1. Evaluation of the models……………………………………………………………………………………………. 3

1.1.2. Assessment of covariates….............................................................................................. 3

1.1.3. Difference in BrainPAD between ExpMed and meditation-naive CUOA……………............ 3

1.1.4. Links between BrainPAD and the accumulated hours of meditation practice in
OldExpMed..……………………………………………………………………………………………………………………………….3

1.1.5. Links between BrainPAD and cognitive and affective regulation capacity measures in ExpMed……………………………………………………………………………………………………………………………………… 4

1.1.6. Longitudinal Impact of the 18-month meditation training in the CUOA………………………. 4

1.1.7. Identification of the brain regions subtending brain aging prediction…………………………. 4

1.2. BrainPAD analyses with age included as a covariate………………………………………………………….4

2. Supplementary Methods………………………………………………………………………….……………………………. 6

2.1. Interventions……………………………………………………………………………………….…………………………… 6

2.1.1. Overview…………………………………………………………………………………….………………….. …………. 6

2.1.2. Meditation training…………………………………………………………………………………….………………. 6

2.1.3. Non-native language training……………………………………………………………………………………… 7

2.1.4. No intervention………………………………………………………………………………………………………….. 8

2.2. Description of tests and questionnaires used for the assessment of the cognitive and affective regulation capacity links to BrainPAD……………………………………………………………………..…………………. 8

2.2.1. Cognitive tests…………………………………………………………………………………….……………………… 8

2.2.2. Affective regulation capacity questionnaires………………………………..…………………………….. 10

2.3. Brain Age Prediction Replication model……………………………………………………………….. …………. 13

2.4. Supplementary Figures………………………………………………………………………………..…………………… 14

2.4.1. Supplementary Figure S1: Evolution of the predicted brain age in relation to the chronological age in the testing sample, as a reflect of model performance (a) of the main model and (b) of the replication model…………………………..…………………..…………………………..…………………………..14

2.4.2. Supplementary Figure S2: Flow chart of the older expert meditators…………………………..15

2.4.3. Supplementary Figure S3: Flow chart of the older meditation-naive controls………………16

2.4.4. Supplementary Figure S4: CONSORT diagram………………………………………………………………17

2.4.5. Supplementary Figure S5: Distribution of accumulated hours of meditation practice in older ExpMed………………………………………………………………………………………………………………………………………18

2.4.6. Supplementary Figure S6: Comparison of BrainPAD between OldExpMed and CUOA with age included as a covariate………………………………………………………………………………………………………...18

2.4.7. Supplementary Figure S7: Associations between BrainPAD of OldExpMed and their accumulated hours of meditation practice with age included as a covariate……………………………...19

2.5. Supplementary tables……………………………………………………………………………………………………….20

2.5.1. Supplementary table S1: Stepwise linear regressions showing the cognitive and affective regulation capacity measures most strongly associated with BrainPAD in OldExpMed with age included as a covariate……………………………………………………………………………………………………………….

3. Supplementary References…………………………………………………………………………………………………. 21

**1. Supplementary results:**

1.1. Replication model

1.1.1 Evaluation of the model

The replication model was able to accurately predict age in the training dataset, based on GMV and WMV maps (predicted-chronological age correlation: p < 0.001, β= 0.95) (Fig. 1). The MAE was 5. This allowed us to validate our model's ability to predict brain age using neuroimaging data in the test dataset, which included ExpMed, as well as CUOA, both pre- and post-intervention. The model was also able to predict chronological age in the test dataset (predicted-chronological age correlation: p < 0.001, β = 0.46, MAE = 5.03).

1.1.2. Assessment of covariates

Sex and education significantly differed between ExpMed and meditation-naive CUOA (sex: p = 0.03, x² = 4.48 ; male/female frequencies – OldExpMed: 16/9, CUOA: 52/82 & education: p = 0.012, t = 2.67 ; mean years of education – OldExpMed: 15.16, CUOA: 13.18) while there was no significant difference for age (p = 0.29, t = 1.09 ; mean age – OldExpMed: 70.3, CUOA: 69.25). Moreover, there was no significant interaction between groups and any of the covariates on BrainPAD (age: p = 0.33, F = 0.95; sex: p = 0.58, F = 0.31; education: p = 0.73, F = 0.12). As a result, sex and education were both included as covariates in the ExpMed *vs* CUOA comparison analysis. Additionally, no significant association was found in ExpMed between BrainPAD and age (p = 0.18, β = -0.44) and education (p = 0.98, β = -0.01) nor was there any difference in BrainPAD between the sexes (p = 0.093, β = -0.27). Therefore, no covariate was included in the regression analyses within the ExpMed group.

1.1.3. Difference in BrainPAD between ExpMed and meditation-naive CUOA

ExpMed had a significantly lower BrainPAD compared to meditation-naive CUOA (p = 0.004, F = 8.15). The between-group difference is illustrated in Fig. 2B in the core article.

1.1.4. Links between BrainPAD and the accumulated hours of meditation practice in ExpMed

BrainPAD was significantly associated with the accumulated hours of meditation practice in ExpMed, showing reduced BrainPAD with greater meditation practice (p = 0.029, β = -0.44) (Fig. 3B).

1.1.5. Links between BrainPAD and both cognitive and affective regulation capacity measures in ExpMed

Results of the stepwise regression are reported in Table 2. As regard to cognitive performance, BrainPAD showed the strongest association with 2D MRT time (mental imagery: p = 0.005, β = 0.59). For emotional processes measures, none of the variables were retained in the final stepwise regression model.

1.1.6. Longitudinal Impact of the 18-month meditation training in the CUOA

The linear mixed model revealed no within-group changes nor significant between-group differences on BrainPAD (F_interaction_ = 0.14, p_interaction_ = 0.86), meaning that the meditation training showed no significant effect on BrainPAD within the group during the intervention nor compared to the other groups (Table 3 and Fig. 4B).

1.1.7. Identification of the brain regions subtending brain aging prediction

For GMV, BrainPAD was negatively associated with voxels in the frontal and temporal lobes, notably in the insula, medial prefrontal cortex, and posterior cingulate cortex. For WMV, BrainPAD was negatively linked to voxels in frontal and temporal tracts such as the corpus callosum, the anterior corona radiata and the cingulum (Fig. 5).

1.2. BrainPAD analyses with age included as a covariate

In the main model, the difference in BrainPAD between OldExpMed and meditation-naïve CUOA was no longer significant after controlling for age (F = 0.73, p = 0.39), so that no further within-group analyses were conducted.

In the replication model, the difference in BrainPAD between OldExpMed and meditation-naïve CUOA remained significant after controlling for age (F = 7.33, p = 0.008; Supplementary Fig. S6). Within OldExpMed, BrainPAD continued to show a significant negative association with accumulated hours of meditation practice across the lifespan when correcting for age (ß = -0.47, p = 0.022; Supplementary Fig. S7). In the stepwise regression analyses, the only cognitive measure retained was 2D MRT time (mental imagery), which remained positively associated with BrainPAD after age adjustment (ß = 0.51, p = 0.019), while no affective regulation measure was retained in the final model (Supplementary table S1).

**2. Supplementary Methods**

2.1. Interventions^1^

*2.1.1. Overview*

The 18-month intervention period starts just after the randomization step for each of the three cohorts. During the study, participants are strongly encouraged not to practice the activity proposed in the other arms (groups). The number of teachers per class and their level of expertise are equal in both interventions. Participants are encouraged to engage in all activities throughout the entire intervention period (i.e., 18 months).

For both the meditation and the foreign language training interventions, each weekly group session is divided into three parts: presentation of a theme, sharing, and formal practice. The first two sessions of each month include an equal share of these three parts (3x40min), session 3 includes more sharing (30/60/30min), while session 4 focuses more on practice (30/30/60). For both interventions, participants benefit from media (manual and audio) for their practice. The media (text, images, audio, video) and activities (alone, in pairs, in groups) rotation help to maintain interest and motivation. In addition, participants have to complete daily practice 20 runlogs at home and throughout the intervention on an electronic tablet to inform on their practice (duration, nature, difficulty and pleasantness level).

Monthly meetings are organized between the scientific investigators and the teachers of both interventions to optimize the intervention monitoring, and homogeneity. The teachers in charge of the intervention can also contact the scientific investigators at any time to keep them informed about any aspects related to the intervention or to the participants, to ensure that the care and follow-up of the participants are optimal.

*2.1.2. Meditation training*

The teaching content of the meditation intervention is shaped in 9 months dedicated to the teaching of mindfulness meditation followed by 9 months dedicated to the teaching of the meditation on loving kindness and compassion. A new educational theme is introduced each month during the first session and is further developed, practiced, and discussed in the other sessions of the month. Mindfulness, or attentive presence, consists of cultivating a vigilant awareness of one's own thoughts, actions, emotions and motivations. The participant learns to intentionally pay attention to their internal or external experiences in the present moment, without making any value judgment. The positive mental states (mental calm, compassion) or negative mental states (ruminations, difficult emotions) are observed without identifying or being absorbed by these experiences. The aim is that the present moment is lived in a more open and flexible way and is less dominated by mental conditioning that is a source of suffering. The mindfulness portion of this program is directly adapted from an 8-month mindfulness-based intervention especially designed for older adults and validated on a group of Francophone older adults.

The practice of kindness-based meditation (short for loving-kindness and compassion meditations) is aimed at improving the relationship with oneself and to the world by addressing in a more positive perspective emotions such as shame, self-criticism, or anger, and by developing gratitude and appreciation for positive experiences such as caring love or compassion. Building on the non-judgmental monitoring capacity developed in mindfulness meditation, the participants will learn to cultivate self- acceptance and kindness toward oneself for instance in relation to one's negative thoughts, distractions, difficult emotions, unpleasant physical sensations to foster appreciation toward positive qualities of one's mind (joy, contentment, …). The participants then learn to extend a similar attitude of care and loving-kindness toward their loved ones, toward neutral persons (e.g. stranger), or toward difficult persons, ultimately recognizing that the need for comfort, security, and happiness is shared by all living beings. There is one day of meditation with about 5 hours of practice during which participants immerse themselves more intensively into meditation practices.

*2.1.3. Non-native language training*

A positioning test is proposed during the inclusion visit at the end of the diagnostic battery

(Table 1) to allow a precise assessment of the initial level of each participant. If the number of participants in the foreign language training group is higher than 15, subgroups of levels based on this test are established to facilitate teaching. The intervention consists of English exercises designed to reinforce each participant’s abilities in understanding, writing, and speaking. Sessions are held by mixing oral comprehension and expression activities to prioritize the acquisition of new vocabulary and new grammatical structures. A large place is given to the recognition of the concepts discussed in previous courses. The progress of the participants is evaluated according to a training follow-up document and personalized or group-oriented help as needed. A day of practice on the Anglo-Norman island of Jersey is organized where participants have a mission to accomplish with information to obtain on different places, objects to find and items to buy.

*2.1.4. No intervention*

Participants in the no intervention group are requested not to change their habits and continue living as they used to before engaging in the study and until its completion. They are specifically asked not to engage in meditation or foreign language training.

2.2. Description of tests and questionnaires used for the assessment of the cognitive and affective regulation capacity links to BrainPAD

*2.2.1. Cognitive tests*

Global cognition was measured by the Mattis Dementia Rating Scale^2^ (DRS), a standardized scale used as a dementia screening instrument which quantifies neuropsychological deficits in individuals with cognitive difficulties. It consists of five subscales (Initiation/Perseveration, Construction, Conceptualization, Memory, and Attention) and a total score ranging from 0 to 144. A higher score indicating greater global cognition.

The D2 selective attention test^3^ was used to evaluate an individual attention and concentration performance. The test consists in finding and crossing the letter “d” accompanied by two lines among distracting elements such as other similar letters or letters “d” with less or more than two lines. Performance was measured using the total of correct answers.

The Trail-Making Test^4^ (TMT) consists in two parts: part A measures visual attention and psychomotor speed by connecting numbers from 1 to 25 in ascending order as quickly as possible, while part B measures executive task switching by alternatively and sequentially connecting numbers and letters (e.g. 1-A-2-B, etc.). The time taken to finish the test is recorded. To measure executive functioning specifically, we subtracted the time to complete part A from the time taken to complete part B.

The Stroop test^4^ allows for the measurement of another aspect of executive functioning: inhibition. The test is separated in three conditions where the participant must: 1) name color samples (red, green or blue in a 10×10 matrix), 2) read aloud color names printed in black, and 3) name the ink’s color of color words. Performance was measured by the time taken to complete the three conditions of the test. We used a score obtained by subtracting the completion time for the first condition from the completion time for the third condition to assess inhibition.

The digit span backward task^5^ was used to evaluate working memory by making the participant repeat an increasingly longer sequence of numbers in reverse order (e.g. 7-1 must be said as 1-7). The test continues as long as the participant responds correctly (up to 16 digits). Performance was measured using the greatest sequence repeated (score ranging from 0 to 16).

Episodic memory was measured using the episodic task of the Autobiographical fluency task^6^. The participant must recall as many events as possible that occurred to him during two periods: between the ages of 20 and 30 and this last year. Performance was evaluated based on the total number of events recalled.

Mental imagery was measured by the 2D-Mental rotation test^7^. It is a computerized test that requires the participant to mentally represent and manipulate (rotate) an object in their mind. From a target object, the participant must pick among 4 differentially-oriented objects the one identical to the target. Performance was assessed using the total number of correct answers (from 0 to 16) and the total completion time.

Verbal learning and memory was assessed using the Wechsler Memory Scale-Revised^5^ (WMS-R). The participant is told a short story, from which he must recall as many elements as possible. Recall is done immediately after the story and once again 30 minutes after to measure both short- and long-term memory. Performance was measured based on the total number of elements recalled (score ranging from 0 to 25).

Verbal memory was also assessed using the California Verbal Learning Test^8^ (CVLT-II). The test requires participants to learn 16 words and to recall them in 5 trials then again after learning an interference list of 16 new words. We used both short- and long-term recall (free and cued) to assess verbal memory. Performance was evaluated based on the number of objects recalled (score ranging from 0 to 16).

Verbal fluency was evaluated by two tests measuring two types of fluency (letter and category^4^). For letter fluency, participants must name as many words beginning with a specified letter (P) as possible in a limited time (2 minutes). For category fluency, the participant must name as many words as possible of a specified category (animal words) in two minutes. In both tests, performance was assessed based on the total number of words (unique eligible answers) enunciated.

*2.2.2. Affective regulation capacity questionnaires*

Mindfulness was measured using the Five-Facet Mindfulness questionnaire^9^ (FFMQ, scores ranging from 15 to 75) which assesses 5 facets of mindfulness (observing, describing, non-judging of inner experience, non-reactivity to inner experience, and acting with awareness). We used the total score which is the sum of the five subscales. This measure uses a 5-point Likert scale ranging from 0 (never or rarely) to 5 (very often or always true), a higher score indicating higher mindfulness.

Body awareness was evaluated using the Multidimensional Assessment of Interoceptive Awareness scale^10^ (MAIA, scores ranging from 1 to 5) which assesses 8 concepts, namely, noticing, not distracting, not worrying, attention regulation, emotion awareness, self-regulation, body listening, and trusting. We used the total score which is calculated by averaging the score from the eight concepts. The Multidimensional Assessment of Interoceptive Awareness scale uses a 5-point Likert scale ranging from 0 (never) to 5 (always), a higher score relating to greater body awareness and interoceptive awareness.

Compassion was measured by the Self-Compassion Scale-Short Form^11^ (SCS-SF, scores ranging from 12 to 60) which is designed to assess typical actions toward one's self in difficult times. This scale measures several aspects of compassion towards oneself such as self-indulgence, self-judgment or mindfulness. This measure uses a 5-point Likert scale ranging from 1 (almost never) to 5 (almost always), a higher score indicating higher self-compassion.

Compassion for others was assessed by the Compassion Towards Other^12^ questionnaire (scores ranging from 21 to 147), a questionnaire designed to measure compassionate love for humanity. Compassionate love for humanity involves behaviors, feelings and thoughts that focus on concern, caring and support for humanity, as well as a motivation to understand and help humanity (strangers) when they are most in need. This measure uses a 7-point Likert scale ranging from 1 (not true for me) to 7 (very true for me), a higher score indicating higher compassion.

Defusion was evaluated using the Drexel defusion scale^13^ (scores ranging from 0 to 50), a questionnaire designed to assess defusion, a state of mind wherein one achieves psychological distance of their thoughts and feelings from subjective experiences. This scale uses a 6-point Likert scale ranging from 0 (not at all) to 5 (perfectly), a higher score relating to higher defusion.

Anxiety was measured using the State Trait Anxiety Inventory Form Y^14^ (STAI, scores from 20 to 80) and is divided in 2 subscales: STAI B assessing the long-standing quality of "trait anxiety" and STAI A measuring the temporary condition of "state anxiety". The essential qualities evaluated by the STAI-Anxiety scale are feelings of apprehension, tension, nervousness, and worry. This measure uses a 4-point Likert scale ranging from 0 (No) to 3 (Yes), a higher score relating to higher levels of anxiety.

Depression was assessed by the Geriatric Depression Scale^15^ (GDS, scores from 0 to 15) and the Death Depression Scale^16^ (DDS, scores from 21 to 105). The GDS is a screening tool for measuring depression in the elderly while the DDS measure depression sadness or somber reflections associated with death. The total score of both scales was used. The GDS is answered by yes/no replies. The death depression scale uses a 5-point Likert scale ranging from 1 (strongly disagree) to 5 (strongly agree). A higher score relates to more depressive symptoms or more intense depressive thoughts.

Emotion regulation was assessed using the Emotion Regulation Questionnaire^17^ (ERQ, scores from 4 to 28 for suppression and from 6 to 42 for cognitive reappraisal), divided in 2 sub-scales: cognitive reappraisal, which measures a cognitive change strategy that involves reinterpreting emotional situations and expressive suppression, which measures a response modulation strategy that involves restricting behavioural displays of emotion. The reappraisal subscale is thought to reflect a positive strategy towards emotional balance while suppression is considered a non-efficient or even detrimental strategy^17^. This measure uses a 5-point Likert scale ranging from 1 (strongly disagree) to 5 (strongly agree), a higher score indicating higher use of each emotional regulation strategy.

Worry was measured using the Penn State Worry Questionnaire^18^ (PSWQ, scores ranging from 16 to 80). The Penn State Worry Questionnaire uses a 5-point Likert scale ranging from 1 (not at all typical of me) to 5 (very typical of me). We used total worry as it represents pervasive negative thoughts and is intricately linked to mood disorders. The total worry score is derived by summing all the respective item scores with higher scores indicating higher levels of worry.

Rumination was measured using the Rumination Response Scale^19^ (RRS, scores ranging from 22 to 88). The Rumination Response Scale describes responses to depressed mood that are self-focused, symptom-focused and focused on the possible consequences and causes of the mood and can be divided in 2 subscales: reflective rumination and brooding rumination. The Brooding subscale (scores ranging from 5 to 20) involves anxious or gloomy thinking is thought to be maladaptive, and is associated with depression^19^. The RRS uses a 4-point Likert scale ranging from 1 (almost never) to 4 (almost always). The Brooding rumination score is derived by summing all the respective item scores with higher scores indicating higher levels of brooding.

Well-being was assessed using the Ryff 7-item Well-Being Scale^20^ (scores ranging from 1 to 7). This scale uses a 7-point Likert scale ranging from 1 (strongly disagree) to 7 (strongly agree), a higher score indicating higher well-being.

Prosocial behavior was measured using the Prosocialness scale^21^ (score ranging from 1 to 80) which assess the four fundamental aspects of prosocialness: behaviors of helping, sharing, taking care of, and feeling empathic with others. The Prosocialness scale uses a 5-point Likert scale ranging from 1 (never/almost never) to 5 (always/almost always). A higher score indicating more prosocial behavior.

Loneliness was evaluated through the Three-item Loneliness Scale^22^ (scores ranging from 3 to 9). This scale uses a simplified set of response categories to measure overall loneliness and was developed for use on the telephone but can be used effectively in person. The Three-item Loneliness Scale uses a 3-point Likert Scale ranging from 1 (almost never) to 3 (often).

2.3. Brain age Prediction Replication model

For the replication model, we used a model developed by the Structural Brain Mapping Group to predict cerebral age^23^. In contrast to the main model – custom-coded and trained on 320 healthy subjects from ADNI – the replication model was externally sourced and was pre-trained on a larger, multi-cohort dataset spanning the entire adult lifespan. Specifically, the replication model was trained with data from 2271 healthy individuals from 5 independent and publicly available neuroimaging initiatives: OASIS (Open Access Series of Imaging Studies), IXI (Information eXtraction from Images), CAMcan (CAMbridge Center for Aging and Neuroscience), SALD (Southwest University Adult Lifespan Dataset), and NKIe (enhanced Nathan Kline Institute) (age range: 30-97.2; mean = 58.83; sd = 14.63; 52% males). Information about data acquisition of these five datasets is available in the following section. The model was trained and developed with MRI images (GMV and WMV maps) preprocessed using the Computational Anatomy Toolbox version 12 (CAT 12) pipeline (<https://neuro-jena.github.io/cat12-help/#long>). The replication model used Gaussian Process Regression (GPR) for brain age prediction. GPR is a machine learning extension of the classical regression model, incorporating nonlinear and Gaussian probabilistic elements to enable quantitative predictions using continuous variables^24^. We thus applied this pipeline to the Age-Well images. After training, the model was applied to the Age-Well images to obtain a predicted brain age.

2.4. Supplementary Figures

**
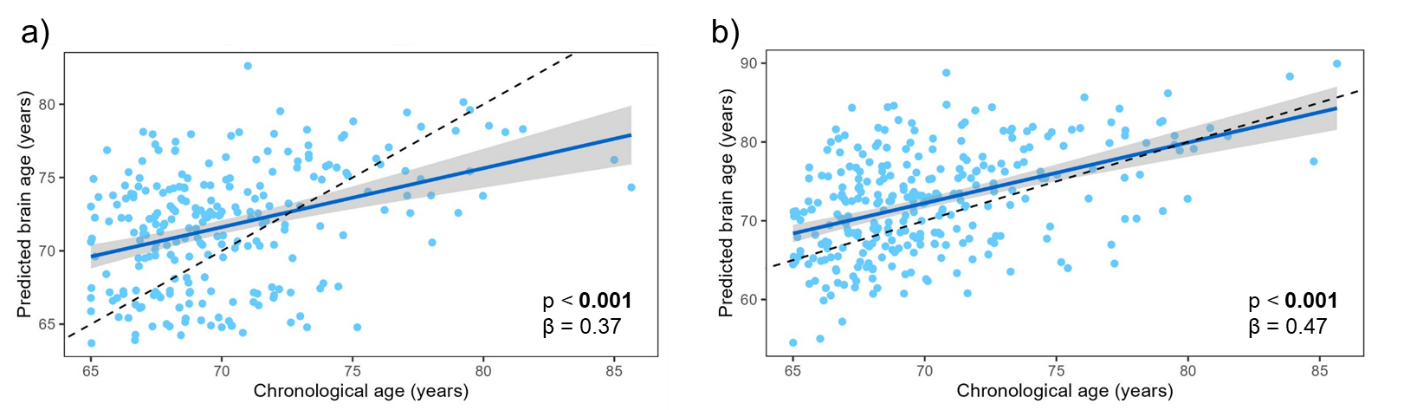
Supplementary Figure S1:** **Evolution of the predicted brain age in relation to the chronological age in the testing sample, as a reflect of model performance (a) of the main model and (b) of the replication model.** *The scatter plots show the relationship between the chronological age and the prediction estimated by the models. Solid lines represent estimated regression lines, dotted lines represent an equal relationship between the chronological age and the predicted brain age, and β represents the slope of the estimated regression.*

**
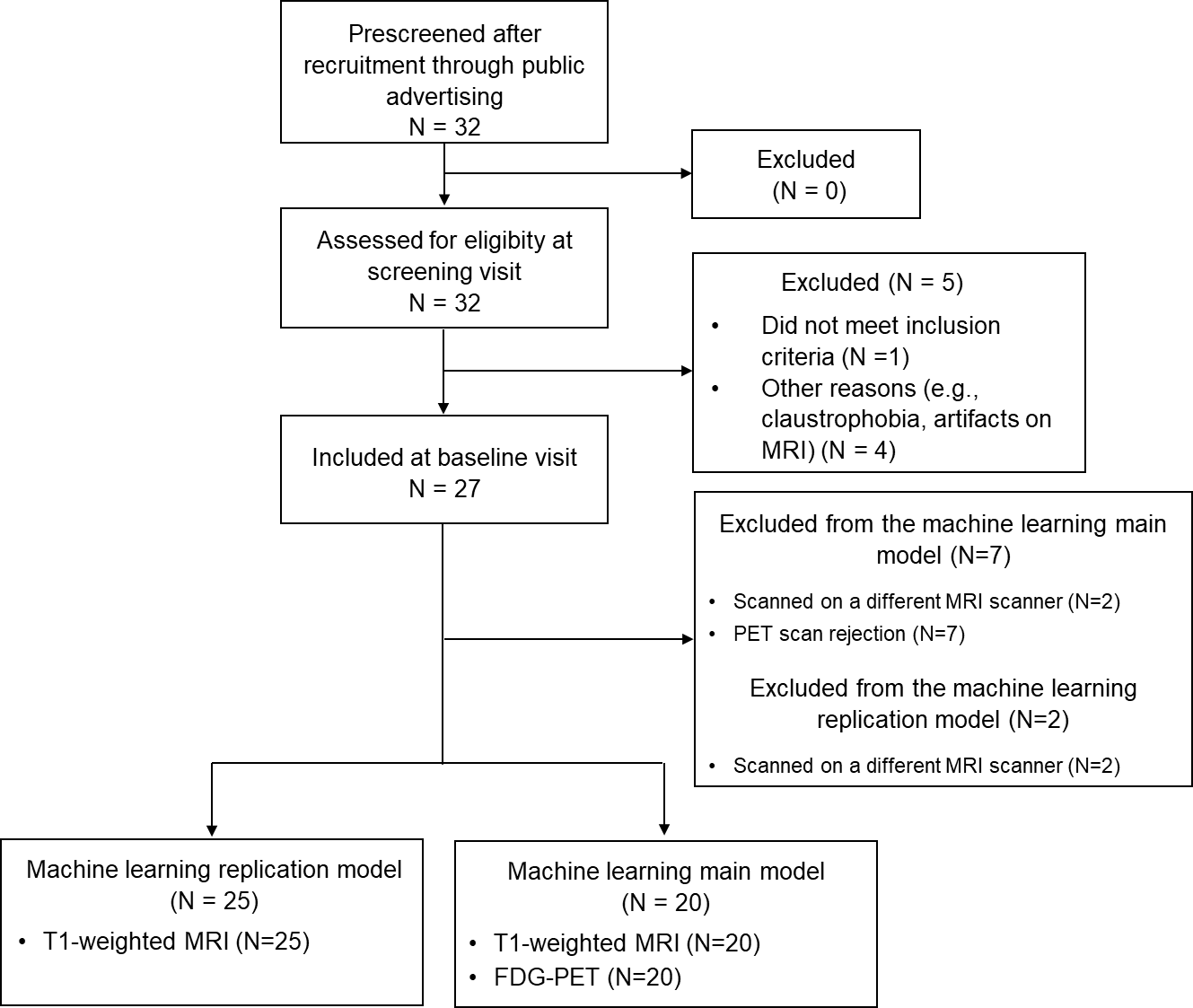
2.4.2. Supplementary Figure S2: Flow chart of the older expert meditators**

**
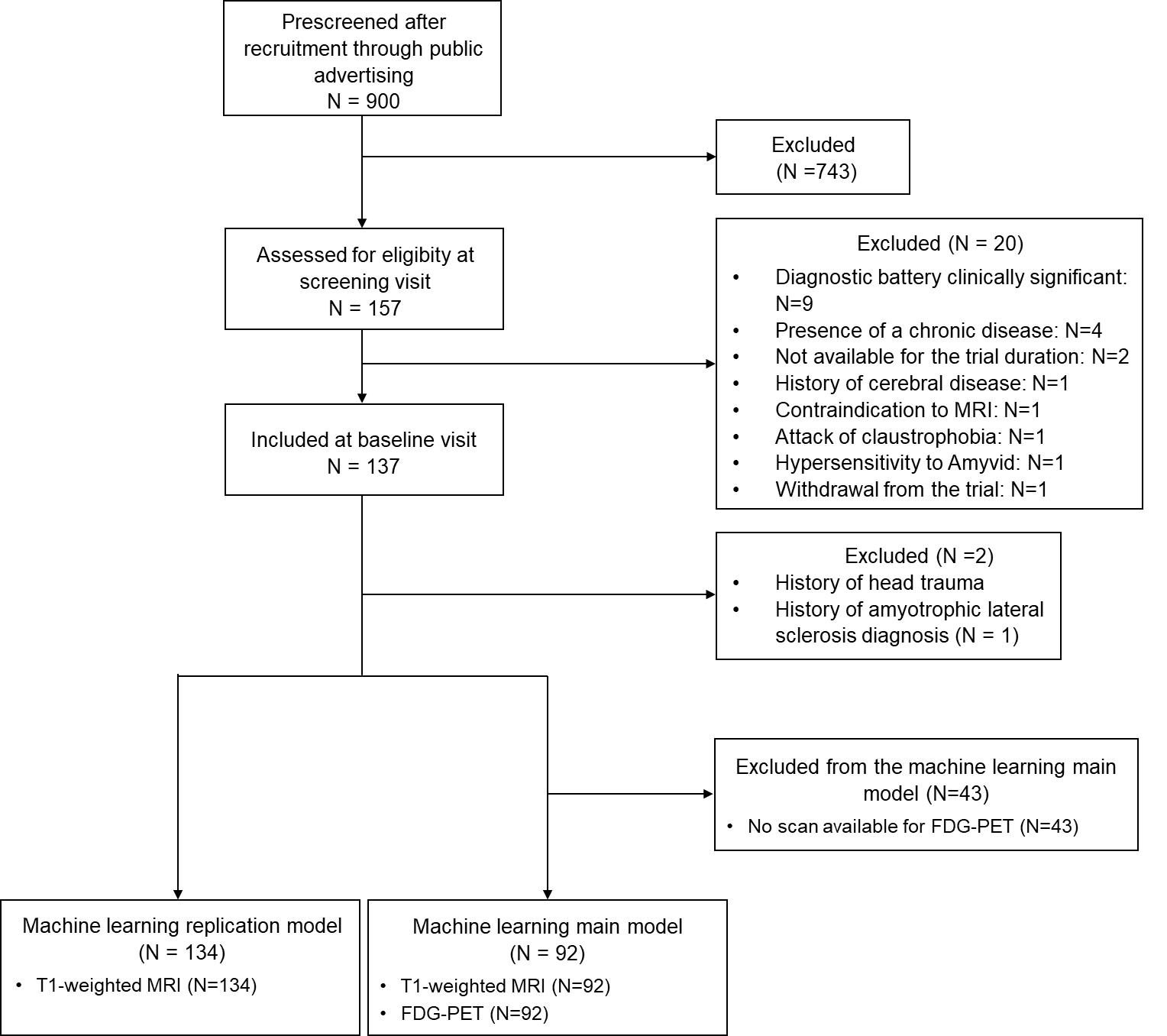
2.4.3. Supplementary Figure S3: Flow chart of the older meditation-naive controls**

**
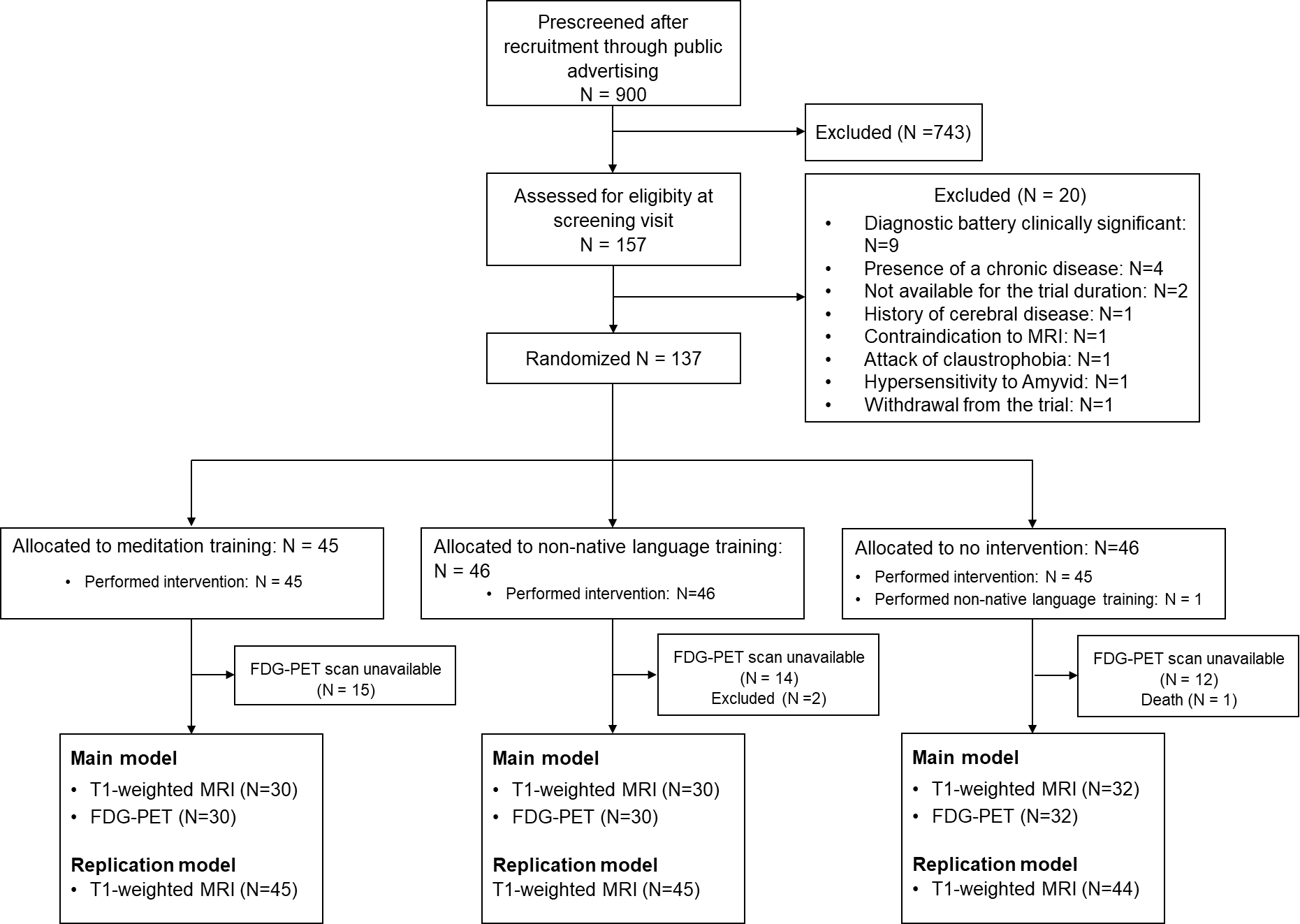
2.4.4. Supplementary Figure S4: CONSORT diagram**

**
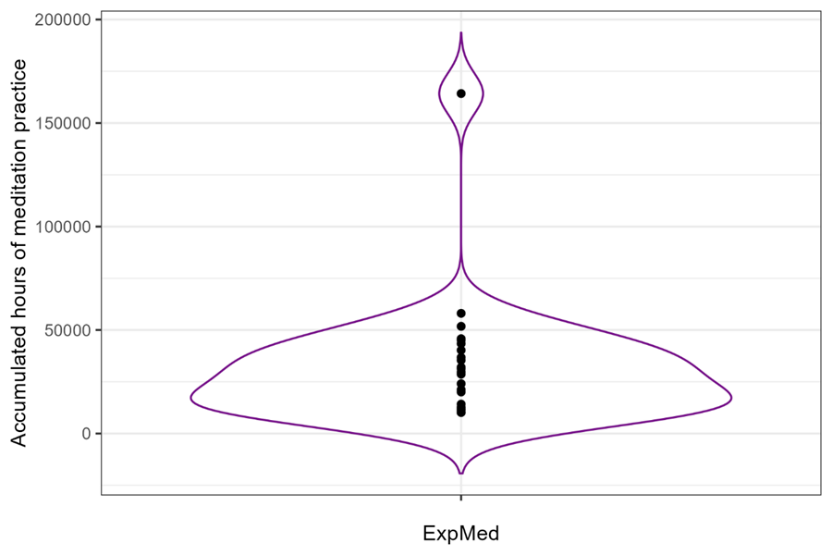
2.4.5. Supplementary Figure S5: Distribution of accumulated hours of meditation practice in older ExpMed.** The violin plot represents the distribution of accumulated hours of meditation practice in the ExpMed group. The spread of the data, shown with the outline, captures the variability of meditation experience, with individual data points plotted to indicate specific practice values. One ExpMed was labelled as an outlier and therefore excluded Abbreviations: ExpMed = Expert Meditators.

**
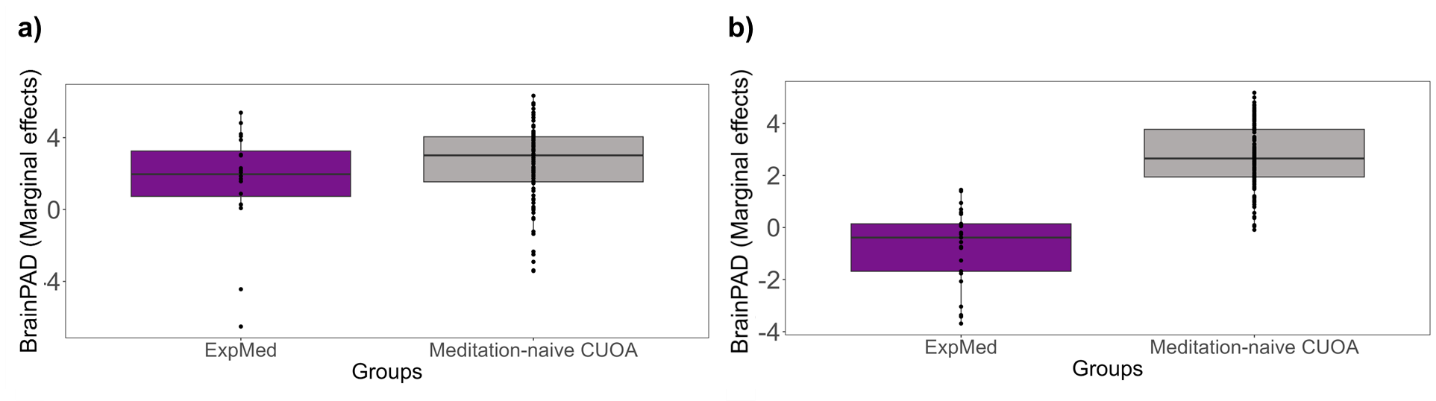
2.4.6. Supplementary Figure S6: Comparison of BrainPAD between OldExpMed and CUOA with age included as a covariate.** The boxplots show the difference of BrainPAD between OldExpMed (purple) and meditation-naive CUOA (gray) (a) with the main model and (b) with the replication model. Marginal effects were plotted to adjust for the effects of age, sex and education. Horizontal lines represent the median of each group, open boxes show the 25^th^ and 75^th^ percentile, and vertical lines show data range. Abbreviations: BrainPAD = Brain Predicted Age Difference, OldExpMed = Older Expert Meditators, CUOA = Cognitively Unimpaired Older Adults.


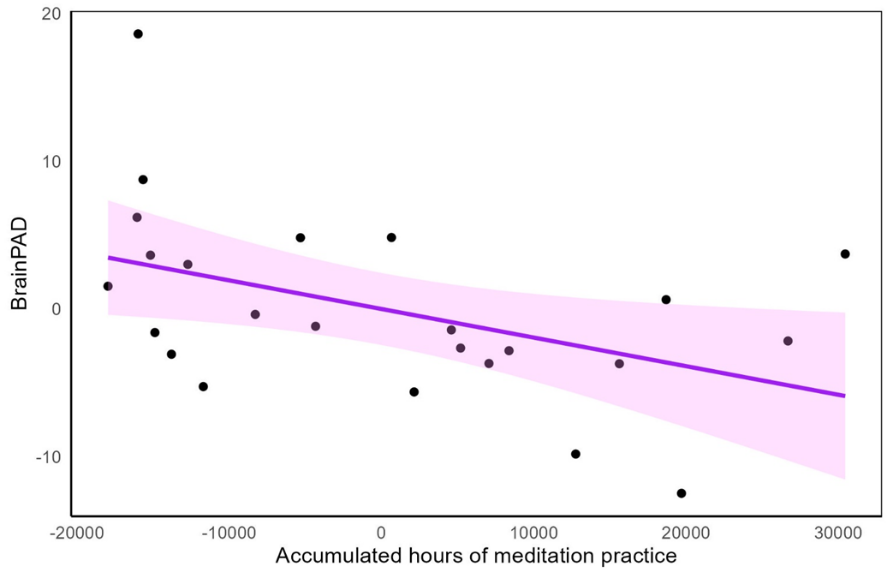
**2.4.7. Supplementary Figure S7: Associations between BrainPAD of OldExpMed and their accumulated hours of meditation practice with age included as a covariate.** The scatter plots show the relationship between the accumulated hours of meditation practice and BrainPAD. Residuals were plotted to adjust for the effects of age. Solid lines represent estimated regression lines, shaded areas represent 95% CI and β represents the slope of the regression. Abbreviations: BrainPAD = Brain Predicted Age Difference, OldExpMed = Older Expert Meditators, CI = Confidence Interval.

2.5. Supplementary tables

**2.5.1. Supplementary table S1:** **Stepwise linear regressions showing the cognitive and affective regulation capacity measures most strongly associated with BrainPAD in OldExpMed with age included as a covariate.**

| **Replication model** | **Coefficient [95% CI]** | ***p*-value** |
| --- | --- | --- |
| **BrainPAD ~** 2D-MRT Time | 0.51 [0.10, 0.93] | **0.019** |

*Abbreviations: BrainPAD = Brain Predicted Age Difference, OldExpMed = Older Expert Meditators, CI = Confidence Interval, 2D-MRT = 2D-Mental Rotation Test (for mental imagery).*

**3. Supplementary References**

1. Poisnel, G. *et al.* The Age-Well randomized controlled trial of the Medit-Ageing European project: Effect of meditation or foreign language training on brain and mental health in older adults. *Alzheimers Dement. N. Y. N* **4**, 714–723 (2018).

2. S, M. Mental Status Examination for Organic Mental Syndrome in the Elderly Patient. *Geriatr. Psychiatry* (1976).

3. Brickenkamp, R. & Zillmer, E. *The D2 Test of Attention*. (Hogrefe & Huber, Seattle, 1998).

4. Godefroy, O. & GREFEX. *Fonctions Exécutives et Pathologies Neurologiques et Psychiatriques*. (2008).

5. Wechsler, D. *WAIS-IV: Wechsler Adult Intelligence Scale*. (Pearson, San Antonio, Tex., 2008).

6. Piolino, P., Desgranges, B., Benali, K. & Eustache, F. Episodic and semantic remote autobiographical memory in ageing. *Mem. Hove Engl.* **10**, 239–257 (2002).

7. Shepard, R. N. & Metzler, J. Mental rotation of three-dimensional objects. *Science* **171**, 701–703 (1971).

8. Delis, D. C. *CVLT-II, California Verbal Learning Test: Adult Version : Manual*. (Psychological Corporation, San Antonio, Tex., 2000).

9. Baer, R. A., Smith, G. T., Hopkins, J., Krietemeyer, J. & Toney, L. Using self-report assessment methods to explore facets of mindfulness. *Assessment* **13**, 27–45 (2006).

10. Mehling, W. E. *et al.* The Multidimensional Assessment of Interoceptive Awareness (MAIA). *PloS One* **7**, e48230 (2012).

11. Raes, F., Pommier, E., Neff, K. D. & Van Gucht, D. Construction and factorial validation of a short form of the Self-Compassion Scale. *Clin. Psychol. Psychother.* **18**, 250–255 (2011).

12. Sprecher, S. & Fehr, B. Compassionate love for close others and humanity. *J. Soc. Pers. Relatsh.* **22**, 629–651 (2005).

13. Forman, E. M. *et al.* The Drexel defusion scale: A new measure of experiential distancing. *J. Context. Behav. Sci.* **1**, 55–65 (2012).

14. Spielberger, C., Gorsuch, R., Lushene, R., Vagg, P. & Jacobs, G. *Manual for the State-Trait Anxiety Inventory (Form Y1 – Y2)*. *Palo Alto, CA: Consulting Psychologists Press;* vol. IV (1983).

15. Yesavage, J. A. & Sheikh, J. I. 9/Geriatric Depression Scale (GDS): Recent Evidence and Development of a Shorter Version. *Clin. Gerontol.* **5**, 165–173 (1986).

16. Templer, D. I. *et al.* Death Depression Scale-Revised. *OMEGA - J. Death Dying* **44**, 105–112 (2002).

17. Gross, J. J. Emotion regulation: affective, cognitive, and social consequences. *Psychophysiology* **39**, 281–291 (2002).

18. Meyer, T. J., Miller, M. L., Metzger, R. L. & Borkovec, T. D. Development and validation of the Penn State Worry Questionnaire. *Behav. Res. Ther.* **28**, 487–495 (1990).

19. Treynor, W. & Gonzalez, R. Rumination Reconsidered: A Psychometric Analysis. *Cogn. Ther. Res.* **27**, (2003).

20. Ryff, C. D. & Keyes, C. L. The structure of psychological well-being revisited. *J. Pers. Soc. Psychol.* **69**, 719–727 (1995).

21. Caprara, G. V., Steca, P., Zelli, A. & Capanna, C. A New Scale for Measuring Adults’ Prosocialness. *Eur. J. Psychol. Assess.* **21**, 77–89 (2005).

22. Hughes, M. E., Waite, L. J., Hawkley, L. C. & Cacioppo, J. T. A Short Scale for Measuring Loneliness in Large Surveys: Results From Two Population-Based Studies. *Res. Aging* **26**, 655–672 (2004).

23. Kalc, P., Dahnke, R., Hoffstaedter, F. & Gaser, C. BrainAGE: Revisited and reframed machine learning workflow. *Hum. Brain Mapp.* **45**, e26632 (2024).

24. Rasmussen, C. E. Gaussian Processes in Machine Learning. in *Advanced Lectures on Machine Learning: ML Summer Schools 2003, Canberra, Australia, February 2 - 14, 2003, Tübingen, Germany, August 4 - 16, 2003, Revised Lectures* (eds. Bousquet, O., von Luxburg, U. & Rätsch, G.) 63–71 (Springer, Berlin, Heidelberg, 2004). doi:10.1007/978-3-540-28650-9_4.
